# Identification and Verification of QTL Associated with Frost Tolerance Using Linkage Mapping and GWAS in Winter Faba Bean

**DOI:** 10.3389/fpls.2016.01098

**Published:** 2016-08-04

**Authors:** Ahmed Sallam, Mustapha Arbaoui, Mohamed El-Esawi, Nathan Abshire, Regina Martsch

**Affiliations:** ^1^Department of Genetics, Faculty of Agriculture, Assiut UniversityAssiut, Egypt; ^2^Department of Agronomy and Horticulture, University of Nebraska-LincolnLincoln, NE, USA; ^3^Unit of Genetics, Biotechnologies and Plant Breeding, Department of Production, Protection and Biotechnology of Plants, Hassan II Institute of Agronomy and Veterinary MedicineRabat, Morocco; ^4^Botany Department, Faculty of Science, Tanta UniversityTanta, Egypt; ^5^Division of Crop Biotechnics, KU LeuvenLeuven, Belgium; ^6^Department of Crop Sciences, Georg-August-Univeristät GöttingenGöttingen, Germany

**Keywords:** faba bean, GWAS, QTL mapping, frost tolerance, QTL validation, Synteny

## Abstract

Frost stress is one of the abiotic stresses that causes a significant reduction in winter faba bean yield in Europe. The main objective of this work is to genetically improve frost tolerance in winter faba bean by identifying and validating QTL associated with frost tolerance to be used in marker-assisted selection (MAS). Two different genetic backgrounds were used: a biparental population (BPP) consisting of 101 inbred lines, and 189 genotypes from single seed descent (SSD) from the Gottingen Winter bean Population (GWBP). All experiments were conducted in a frost growth chamber under controlled conditions. Both populations were genotyped using the same set of 189 SNP markers. Visual scoring for frost stress symptoms was used to define frost tolerance in both populations. In addition, leaf fatty acid composition (FAC) and proline content were analyzed in BPP as physiological traits. QTL mapping (for BPP) and genome wide association studies (for GWBP) were performed to detect QTL associated with frost tolerance. High genetic variation between genotypes, and repeatability estimates, were found for all traits. QTL mapping and GWAS identified new putative QTL associated with promising frost tolerance and related traits. A set of 54 SNP markers common in both genetic backgrounds showed a high genetic diversity with polymorphic information content (PIC) ranging from 0.31 to 0.37 and gene diversity ranging from 0.39 to 0.50. This indicates that these markers may be polymorphic for many faba bean populations. Five SNP markers showed a significant marker-trait association with frost tolerance and related traits in both populations. Moreover, synteny analysis between *Medicago truncatula* (a model legume) and faba bean genomes was performed to identify candidate genes for these markers. Collinearity was evaluated between the faba bean genetic map constructed in this study and the faba bean consensus map, resulting in identifying possible genomic regions in faba bean which may control frost tolerance genes. The two genetic backgrounds were useful in detecting new variation for improving frost tolerance in winter faba bean. Of the five validated SNP markers, one (VF_Mt3g086600) was found to be associated with frost tolerance and FAC in both populations. This marker was also associated with winter hardiness and high yield in earlier studies. This marker is located in a gene of unknown function.

## Introduction

Faba bean is primarily grown as a livestock feed in Europe, while, it is used as a food crop in developing countries due to its highly protein content which ranges from 19 to 39% (Maxted and Bennett, 2001). Frost stress is a major abiotic factor, which affects faba bean growth. Faba bean is considered to be one of the most cold-tolerant major grain legumes. However, frost resistance is a limiting factor to faba bean production and productivity in many regions. The faba bean germplasm has different levels of frost tolerance, reaching a maximum in the French cultivar Côte d'Or which can tolerate down to −25°C without snow coverage (Picard et al., 1985). However, faba bean is sown as a spring crop in cool temperate climate countries due to insufficient winter-hardiness and frost tolerance in the current Autumn-sown genotypes (Arbaoui, 2007; Arbaoui et al., 2008a). For instance, the winter season of 2012/2013 in Göttingen, Germany was too severe for the majority of winter beans (nearly zero survival). There were 12 days of freezing temperatures below −11°C, with the minimum temperature at −19.5°C and without snow coverage (Sallam, 2014). In addition, winter faba bean has a higher yield production than spring bean. Therefore, breeding for winter faba bean is urgently needed to produce resistant and high yielding cultivars.

Frost tolerance is a highly heritable trait with large additive effects and is controlled by many genes (Duc and Petitjean, 1995) or quantitative trait loci. The progress in the genetic improvement of winter faba bean germplasm using classical plant breeding programs has been slow. Moreover, the effectiveness of selection was not fruitful due to genotype and environment interaction (Rama et al., 2014). In light of the above difficulties, marker assisted-selection (MAS) paves the way to dissect frost tolerance at the genomic level. Arbaoui et al. (2008b) reported important putative QTL for frost tolerance and fatty acid composition (FAC), which alleviate the effect of frost stress in faba bean leaves at seedling stage. Moreover, a set of 67 putative QTL for physiological and morphological traits associated with frost tolerance in faba bean were reported by Sallam et al. (2015b) using SNP markers. All the aforementioned QTL are putative and cannot be used directly for MAS to improve frost tolerance in winter faba bean.

An essential step in MAS is to validate these putative QTL in order to genetically improve frost tolerance in faba bean. The QTL validation examines whether the same QTL appears when the genetic background is grown in other locations and/or years, and whether its effect can still be detectable when introduced into a different genetic background (Landi et al., 2005). The validation can be tested by using different genetic background with different population production methods [e.g., backcross, recombinant inbred lines, multi-parent advanced generation inter-cross (MAGIC) population]. This will help in unraveling the molecular basis of tolerance to frost, which will provide novel opportunities for more applications such as identifying genes responsible for frost tolerance in faba bean.

Studying model species will allow us to better understand many processes including plant response to biotic and abiotic stresses (Swindell et al., 2007), plant development (van Hengel et al., 2004; De Smet and Jürgens, 2007), and physiological adaptations of plants to threatening stress. *Medicago truncatula*, a sequenced model legume, is useful for researchers due to its small genome size (*M. truncatula* around 500 Mb; Gnanasambandam et al., 2012), which is much better suited to genetic and genomic research than large genomes such as that of *Vicia faba* (around 13.000 Mb, Ellwood et al., 2008). QTL for promising traits could be mapped and utilized in selection programs. Moreover, candidate genes involved in stress tolerance processes and/or quality traits could be useful in developing transgenic lines with tolerance to such stresses (Rispail et al., 2010). The *M. truncatula* seed represents a good model for identifying genes controlling seed composition in grain legumes. These genes can be used to investigate fatty acid and sugar compositions in grain legumes, such as pea and faba bean (Duc, 2004; Djemel et al., 2005). Burstin et al. (2007) mapped some putative QTL for traits controlling vegetative plant development, seed yield and protein content in pea. They revealed that faba bean possesses homologous loci which represented the same trait, and hence such loci could be used in selection schemes. Although Brandsæter et al. (2002) reported that *M. truncatula* reveals a poor freezing tolerance, when compared to other annual legumes. QTL for frost tolerance traits were recently mapped in *M. truncatula* by Avia et al. (2013). This could actually help in identifying resistance genes to frost stress. Some studies reported promising results for low temperature legume breeding in alfalfa using transgenic expression of an iron-superoxide dismutase, resulting in an enhanced winter survival (McKersie et al., 1993). Recently, a faba bean consensus map (FBCM) was developed with six linkage groups which are presumed to have a correspondence to the six faba bean chromosomes (Webb et al., 2015). The FBCM was syntenyed to *M. truncatula* genome. This should provide valuable information on the possible genes controlling important traits in faba bean.

Unfortunately, very few studies has been carried out before on QTL validation for frost tolerance in faba bean (Sallam and Martsch, 2016). Therefore, the objectives of this study were (1) to construct a genetic map for faba bean using recombinant inbred lines (RILs) population and identify QTL for traits associated with frost stress tolerance, (2) to validate some QTL associated with frost tolerance that were previously reported by Sallam and Martsch (2015) in a different genetic background (RILs population), and (3) to identify candidate genes underlying common QTL controlling frost tolerance in both genetic backgrounds using synteny between the *M. truncatula* and faba bean genomes.

## Materials and methods

### Plant material

The plant material of this study consisted of two different genetic background populations:

1. Biparental population (BPP):
  The BPP consisted of 101 F_6_ recombinant inbred lines (RILs) derived from the cross between two frost tolerant parental lines Côte d'Or 1 (French landrace), and BeanPureLine 4628 (BPL4628, Chinese inbred line, Arbaoui et al., 2008b).
2. Gottingen Winter Bean population (GWBP):
  This population consisted of 189 single seed descent (SSD) (>F_9_) which were derived from 11 highly adapted winter beans lines (founder lines, FL). The FL originated from three different parts in Europe: Germany (Webo, Wibo, Hiverna/1, L79/79, L977/88, and L979/S1), France (Côte d'Or/1 and Arrissot), and UK (Banner, Bourdon, and Bulldog). The production of GWBP were previously described in Sallam et al. (2015b).

### Phenotypic evaluation

All experiments of the current study were conducted under controlled conditions in a frost growth chamber (FGCh, size of 2 × 2 × 2 m^3^) at Department of Crop Sciences, Georg-August-Univeristät Göttingen. The seeds of each genotype were sown in pots 17 × 17 × 17 cm^3^ with compost soil and sand (3:1). The irrigation was applied to keep a 70% soil water capacity. In FGCh, light condition was programmed at 200 μmol s^−1^ m^−2^ during 12 h. The air humidity fluctuated between 80 and 90% according to temperature fluctuation. At the seedlings stage, different traits were measured. When all seedlings had reached to two expanded leaves, the pots were transferred to the FGCh.

#### Artificial frost test

Seedlings of BPP and GWBP lines were exposed to different regimes of freezing temperature according to Arbaoui and Link (2007) and Sallam et al. (2015b), respectively.

In BPP, two treatments were applied on the juvenile faba bean plants: hardening (2.5°C days/0°C nights during 1 week) and unhardening conditions. After both treatments, all plants were tested for their frost tolerance through six steps of decreasing freezing temperature (−8, −10, −13, −16, −19, and −21°C) as described in Arbaoui and Link (2007). After each freezing test, each plant/genotype was scored for loss of turgidity (1 = not turgid, 4 = full turgid) and loss of leaf color (1 = green, 4 = black). The genotypes in both treatments were evaluated in three experiments with two replications each (*r* = 6). An area under symptom progress curve (AUSPC; corresponding to the “Area Under Disease Progress Curve,” AUDPC, Shaner and Finney, 1977) was calculated to reflect the symptoms of frost stress on faba bean for each genotype after hardening (H_AUSPC) and unhardening (U_AUSPC) (c.f. Arbaoui and Link, 2007). In addition, the mean value of hardening and unhardening for each genotype (M_AUSPC) was calculated.

For GWBP, two seeds from each genotype were sown in pots (four different genotypes in one pot). The 189 juvenile faba bean plants were exposed to only a hardening (2.5°C days/0°C nights for 10 days) phase before frost test (Sallam et al., 2015b) which was applied for three nights under three freezing temperatures (−16, −18, and −19°C). After each step, each plant/genotype was visually scored for loss of turgidity (1 = full turgid, 9 = not turgid) and loss of color (1 = green, 9 = black). All genotypes were evaluated in 10 experiments with two replications each (*r* = 20). The AUSPC trait (frost tolerance) was calculated as previously mentioned in BPP. After the third step of frost, all plants were given a break of 4 days at 8°C in the green houses. All genotypes were visually scored again for their loss of turgidity after frost (LTAF) and loss of color after frost (LCAF). The detailed steps of frost experiments applied on GWBP were previously described by Sallam et al. (2015b).

#### Physiological traits

##### Fatty acid composition (FAC)

For BPP, the first leaves before and after hardening of each genotype were analyzed for FAC as described in Arbaoui et al. (2008a). The genotypes were randomized in three experiments with two replications each (*r* = 6). FAC was also analyzed in the GWBP but only after hardening conditions in two experiments with two replications in each (*r* = 4) (Sallam et al., 2015b).

##### Free proline content

The free proline content in hardened leaves of BPP was analyzed. Seeds of each genotype were first imbided in water for a couple of days at 21°C. Then, the seeds were sown in vermiculite in 10 × 10 × 10 cm^3^ pots. The growth chamber was programmed at 21°days/18°C nights and the photoperiod was set up for 16 h during germination and emergence. Hoagland solution was used to irrigate the seedlings daily. Consequently, seedling were transferred after 2 weeks to FGCh for hardening treatment (4°C days/2°C nights for 3 weeks). Leaf discs (youngest fully grown leaf) were cut and freeze-dried, dry weight was measured. Free proline content was photometrically estimated according to Troll and Lindsley (1955) as modified by Bates et al. (1973). For every genotype, there were four replicates containing five leaf discs from different plants.

The list of abbreviations of traits is presented in a Supplementary Table (Appendix 1).

#### Statistical analysis of phenotypic data

For all traits scored in both populations, the analysis of variance and repeatability estimates was calculated using Plabstat (Utz, 1991). The statistical analyses of different traits for both populations were extensively described in Arbaoui et al. (2008b) and Sallam et al. (2015b).

### DNA extraction and SNP genotyping

DNA was extracted from the leaves of each genotype using Illustra Nucleon Phytopure Genomic DNA Extraction kits (GE Health UK Limited). The DNA was SNP-genotyped using KASPar™ (Kompetitive Allele Specific PCR) assay platform (KBioscience, UK), a single-plex SNP genotyping methodology using allele-specific amplification followed by application of fluorescence detection for genotyping. A total of 189 single nucleotide polymorphism (SNP) markers were chosen from a set of 687 SNPs that have been mapped in the faba bean consensus map (FBCM) of Webb et al. (2015). Therefore, both populations (BPP and GWBP) were genotyped using the same 189 SNP markers (Table S6). The 189 SNP markers were originally generated from *Medicago truncatula* (a sequenced model legume).

### QTL mapping

The 101 RILs (BPP) were used to perform QTL mapping. The deviation from expected Mendelian segregation ration of 1:1 was assessed using Chi square test (χ^2^) for all the 189 SNP markers. Of the 189 SNPs, 117 showed normal diploid segregation (*P* ≥ 0.05) and were used to construct the genetic map. The genetic linkage map was constructed using MapDisto v. 1.7.7.0.1 (Lorieux, 2012) with a logarithm of odds (LOD) score of 3.0 and recombination fraction of 0.3. The genetic map distance (centiMorgans; cM) was calculated based on the Kosambi function (Kosambi, 1943). The QTL mapping was performed using mixed linear composite interval mapping (CIM) method to find the relationship between each linkage group and putative QTL location using Network QTL 2.1 (Yang et al., 2008). Composite interval analysis was undertaken using forward–backward stepwise. The genome was scanned at a walk speed of 1 cM. Moreover, a 2D genome scan, a probability into and out of the model of 0.05, and a window size set at 10 cM were applied. QTL detection was estimated with 1000 permutations and a genome-wide error rate of 0.10 (suggestive) and 0.05 (significant) as a significant threshold. Linkage groups and QTL positions were drawn using MapChart v. 2.2 (Voorrips, 2002).

Collinearity analysis was performed between the genetic map constructed in this study and FBCM constructed by Webb et al. (2015) using 687 SNPs (mapped in 6 linkage groups) in order to figure out the locations of our linkage groups in the consensus map of faba bean. The synteny was also drawn using MapChart v. 2.2.

### Genome wide association study (GWAS)

The GWAS was performed in GWBP to study the association among SNP markers and traits scored on the population (AUSPC, LTAF, and LCAF). The analysis was carried out using TASSEL version 5.0 (Bradbury et al., 2007). Of the 189 SNP markers, 156 markers showed a clear polymorphism with a minimum allele frequency of 10% between the 189 genotypes. The association mapping was performed using general linear (GLM) and mixed linear (MLM) + kinship models. The marker-trait association was detected at significant threshold of 0.20 false discovery rate (FDR) as described in Benjamin and Hochberg (1995), Cai et al. (2014), Honsdorf et al. (2010) and Sallam and Martsch (2015).

### Analysis of genetic diversity of GWBP and BPP populations

Polymorphic information content (PIC) and gene diversity of the SNPs markers in GWBP and BPP populations were calculated using PowerMarker software V 3.25 (Liu and Muse, 2005). The PIC value was used to describe the marker value based on the polymorphism level revealed. The PIC value was calculated based on the following formula (Dhanapal et al., 2015):

$$\begin{array}{l} \text{PIC }=\;1-\overset{n}{\underset{j=1}{\sum }}P_{ij}^{2}-\overset{n=1}{\underset{j=1}{\sum }}\overset{n}{\underset{k=j+1}{\sum }}{2P}_{ij}^{2}P_{ik}^{2} \end{array}$$

where *P*_*ij*_ and *P*_*ik*_ indicate the frequencies of *j*_*th*_ and kth alleles for marker *i*, respectively. The gene diversity is described as the probability that two alleles randomly picked out from the test sample are different.

### Synteny and candidate genes

The synteny between SNP markers (marker-trait association) and *Medicago truncatula* genes was investigated. The annotation and gene ontology of the candidate genes with markers, which showed a significant association with frost tolerant traits in both populations, were identified using database LegumeIP (http://plantgrn.noble.org/LegumeIP/) (*M. truncatula*, gene model, Mt3.5v3). Moreover, the maximum rate of gene expression in tissue was identified using the database LegumeIP to recognize the specific tissue (two or three tissues were selected for comparison) in which the gene has the highest expression rate. The tissue with the highest gene expression was used in experiments which did not include any treatment.

## Results

### Genetic variation in frost tolerance and related traits in both populations

The genetic variation for frost tolerance (H_AUSPC and U_AUSPC) and some FAC contents (C16:0, C18:0, C18:1, C18:2, C18:3, and C18:4) of hardened and unhardened plants were already studied by Arbaoui et al. (2008b). In the present study, free proline content (after hardening) and C16:1 content were analyzed (after both hardening and unhardening conditions). High significant genetic differences were found between genotypes for free proline and C16:1 contents (Table S1, Supplementary File). The correlation analysis between frost tolerance and physiological traits scored in BPP is presented in Table S2. The proline content showed a negative significant correlation with H_AUSPC and M_AUSPC. Of fatty acid contents, U_C18:0 and H_C18:0 were found to be positively and significantly correlated with H_AUSPC. U_AUSPC showed significant correlations with H_C16:0 (*r* = −0.21^*^), H_C18:2 (*r* = −0.28^**^), H_C18:1 (*r* = 0.35^**^), and U_C18:0 (*r* = 0.22^*^).

For GWBP, a high genetic variation was found between all genotypes for AUSPC, LTAF, and LCAF. The repeatability estimates ranged from 0.90 for AUSPC and 0.94 for LTAF and LCAF (Table S1). In GWBP, high positive significant correlations (*r* = > 0.84^**^) were found among frost tolerant traits (Figure S1).

### Molecular genetic map

**Figure 1:**
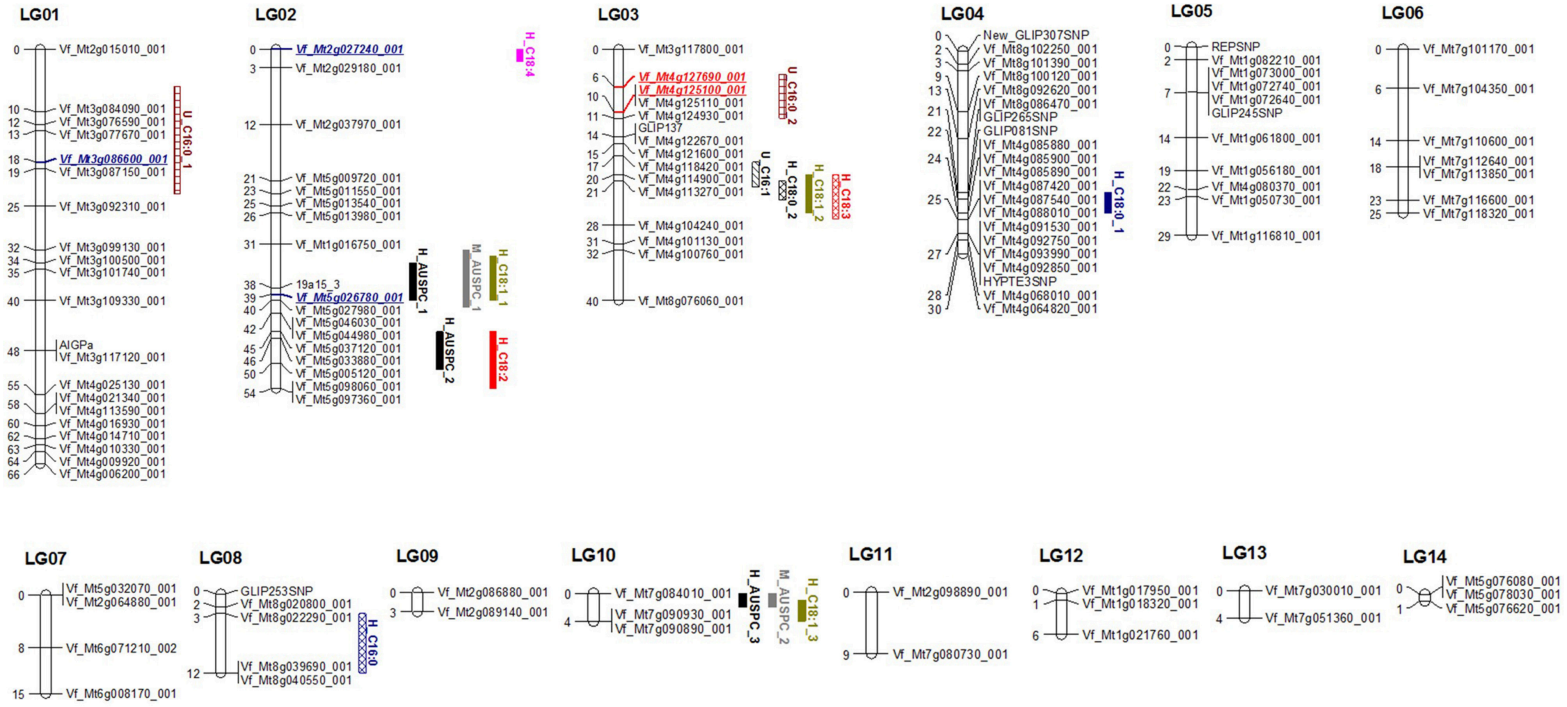
**Winter faba bean genetic linkage map based on 117 SNP markers showing location of QTL for frost tolerance and FAC in 101 RILs**. Red markers refer to validated QTL for the same trait. Blue marker refer to validated QTL for other frost tolerance or/and FAC.

Of the total 189 SNP markers used in screening for polymorphism between the two parents of BPP, 122 (64.6%) showed an agreement with the expected 1:1 segregation ratio using χ^2^ test (*P* ≥ 0.05). On the other hand, the remaining 67 (35.4%) SNP markers showed a significant segregation distortion. The observed direction of the distorted markers for most of the loci was found for the PBL4268 allele. The genetic linkage map (LG) was constructed using 122 SNP markers. Only five SNP markers were not assigned to any linkage group. The rest of 117 SNP markers were mapped to 14 linkage groups. The number of SNP markers in the 14 LGs ranged from 2 to 21 markers (Figure 1, Table S3). The linkage groups constructed in this study were named and numbered from LG01 to LG14. LG01 and LG04 included the highest number of 21 loci covering a map length of 65.97 and 29.61 cM, respectively. The lowest number of loci, on the other hand, was present in LG09, LG11, and LG13 with two markers each and a map length of 2.73, 8.64, and 4.41 cM, respectively. The longest gap in the genetic linkage map was found in LG01 with 10 cM. The average marker interval of the faba bean genetic map constructed in the present study was 3.20 cM.

### Colinearity between genetic map and faba bean consensus map (FBCM)

The collinearity between loci in the current genetic linkage map of BPP and loci mapped in FBCM (Webb et al., 2015), is illustrated in Figure S2 and Table S4. All the 14 LGs were distributed across the six linkage groups of FBCM (FBCM_01, 02, 03, 04, 05, and 06). FBCM_01 had the highest number of collinear LGs, with five (LG02, 07, 09, 11, and 14) and covering a total map length of 80.73 cM, while, one LG (LG01) is collinear with FBCM_02 and another one (LG04) with FBCM_06. Notably, LGs attached with the FBCM were distributed in different positions and there was not a specific cluster for any group of LGs (Figure S2). Not all markers, which were mapped in this study, were mapped in FBCM. Of the 117 marker loci, 104 were found to be mapped in the FBCM. The percentage of the mapped markers in FBCM ranged from 66.6 to 100% (Table S4).

### QTL analyses in BPP and GWBP

The 101 lines (BPP) were used to detect QTL using the full QTL model of QTL Network software (Table 1, Figure 1, and Table S7). Seventeen significant QTL were detected for 11 frost tolerant and physiological traits. The proportion of phenotypic variation explained (PVE) by QTL ranged from 2.74 to 29.41%. The highest number of QTL was found in LG02, having six. Obviously, there was clustering of different QTL in LG02 and LG03. All detected QTL were distributed on six LGs (01, 02, 03, 04, 08, and 10). A significant epistatic interaction was observed only with the QTL for proline content (Table 2, **Figure 3**).

**Table 1:** **QTLs for frost tolerance and fatty acid composition identified by QTL Network 2.1 using biparental population (101 RILs)**.

**Trait**	**QTL**	**Interval**	**LG^a^**	**Position^b^**	**Effects^c^**	***P*-value**	***F*-value**	**PVE^d^**
H_AUSPC	H_AUSPC_1	19A15_3-VF_MT5G026780_001	LG02	38.5	−20.01	0.00321	16.7	18.79
	H_AUSPC_2	VF_MT5G033880_001-VF_MT5G005120_001	LG02	47.8	−22.77	0.00202	20.2	13.15
	H_AUSPC_3	VF_MT7G084010_001-VF_MT7G090890_001	LG10	0.0	−30.38	0.00005	19.3	17.00
M_AUSPC	M_AUSPC_1	VF_MT1G016750_001-19A15_3	LG02	36.3	−28.56	0.00007	16.7	14.79
	M_AUSPC_2	VF_MT7G084010_001-VF_MT7G090890_001	LG10	0.0	−25.77	0.00011	16.7	14.35
H_C16:0	H_C16:0	VF_MT8G022290_001-VF_MT8G039690_001	LG08	8.1	−0.22	0.00050	15.5	10.89
H_C18:0	H_C18:0_1	GLIP081SNP-VF_MT4G085880_001	LG04	23	−0.05	0.00000	28.4	22.27
	H_C18:0_2	VF_MT4G113270_001-VF_MT4G104240_001	LG03	24.5	−0.04	0.00027	15.0	15.04
H_C18:1	H_C18:1_1	19A15_3-VF_MT5G026780_001	LG02	37.5	−0.24	0.00541	13.5	2.74
	H_C18:1_2	VF_MT4G113270_001-VF_MT4G104240_001	LG03	21.5	−0.34	0.00008	17.7	14.06
	H_C18:1_3	VF_MT7G090890_001-VF_MT7G090930_001	LG10	4.1	0.64	0.00000	23.0	29.41
H_C18:2	H_C18:2	VF_MT5G033880_001-VF_MT5G005120_001	LG02	47.8	0.62	0.00004	18.6	14.24
H_C18:3	H_C18:3	VF_MT4G113270_001-VF_MT4G104240_001	LG03	22.5	0.62	0.00021	13.6	12.07
H_C18:4	H_C18:4	VF_MT2G027240_001-VF_MT2G029180_001	LG02	0.0	−0.18	0.00003	17.0	14.83
U_C16:0	U_C16:0_1	VF_MT3G077670_001-VF_MT3G086600_001	LG01	15	0.18	0.00116	11.8	11.38
	U_C16:0_2	VF_MT4G127690_001-VF_MT4G125100_001	LG03	8.3	−0.18	0.00123	12.6	11.28
U_C16:1	U_C16:1	VF_MT4G114900_001-VF_MT4G113270_001	LG03	19.9	0.07	0.00004	17.0	14.55

Traits, H_AUSPC, hardened AUSPC; M_AUSPC, mean of hardened and unhardened AUSPC; H_C16:0, hardened palmitic acid; H_C18:0, hardened stearic acid, H_C18:1, hardened oleic acid, H_C18:2, hardened linoleic acid; H_C18:3, hardened linolenic acid; H_C18:4, hardened stearidonic acid, U_C16:0, unhardened palmitic acid; U_C16:1, unhardened palmitoleic acid.

a Linkage group.

b Positon of in the linkage group.

c QTL effects were calculated as mean of Cote d'Or genotypic class—mean of BPL genotypic class. Positive values refer that Cote d'Or carries the allele which associated with an increase in the trait. Negative values, on the other hand, refer that BPL carries the allele which associated with an increase in the trait.

d Phenotypic variation explained by each QTL.

**Table 2:** **Epistatic interactions between QTLs for proline content measured in BPP**.

**Locus**	**Marker interval**	**Position**	**LG**	**Locus**	**Marker interval**	**Position**	**LG**	***P*-value**	**PVE^a^**
Proline_1	VF_MT8G101390_001-VF_MT8G100120_00	3.6	LG04	Proline_2	VF_MT7G084010_001-VF_MT7G090890_001	0	LG10	0.000000	25.34

a Phenotypic variation explained by QTL.

Genome wide association study (GWAS) was used to detect the QTL for frost tolerance in GWBP. Three morphological traits reflecting the symptoms of frost injury on faba bean seedlings were scored and analyzed. Twenty five significant SNP markers were found to be associated with the three traits AUSPC (after hardening), LTAF, and LCAF (Table 3) using GLM. Two markers Vf_Mt4g007030_001 and Vf_Mt3g086600_001 associated with two traits, AUSPC and LTAF using MLM+kinship. The PVE ranged from 2.66 to 11.89%. All markers were mapped in FBCM except one marker GLIP081SNP which was mapped in the current faba bean genetic map of BPP in LG03.

**Table 3:** **The association analysis for AUSPC, LTAF, and LCAF in GWBP**.

**Trait**	**FBCM^a^**	**Marker locus**	**Allele^b^**	**Position^c^**	***p***	**PVE %^d^**	**Allele Frequency^e^**	**Allele Effects^f^**
H_AUSPC	CM01	**Vf_Mt5g046030_001**	T:C	293.36	0.00045	7.93	0.53	−6.82
	CM01	Vf_Mt5g015280_001	T:C	62.66	0.00164	4.53	0.37	−5.09
	CM01	Vf_Mt2g027240_001	C:T	401.92	0.00230	4.05	0.54	−5.13
	CM02	**Vf_Mt3g086600_001**	G:T	103.65	0.00032	7.19	0.89	−10.96
	CM02	Vf_Mt4g007030_001	C:A	234.93	0.00164	4.54	0.34	−5.43
	CM02	Vf_Mt5g075540_001	G:C	31.06	0.00296	3.55	0.49	−4.53
	CM03	Vf_Mt1g105040_001	G:T	97.13	0.00256	5.22	0.85	−7.68
	CM03	Vf_Mt1g056180_001	A:G	227.32	0.00953	4.23	0.29	−5.44
	CM04	Vf_Mt4g101130_001	T:C	24.50	0.00296	3.42	0.26	−6.92
	CM04	Vf_Mt8g040550_001	A:G	192.4	0.00329	3.03	0.41	−4.26
	LG03	GLIP081SNP	G:C	21.99	0.00632	2.66	0.41	−3.99
LTAF	CM01	**Vf_Mt5g046030_001**	T:C	293.36	0.00001	11.89	0.53	−0.63
	CM01	Vf_Mt2g027240_001	C:T	401.92	0.00193	5.15	0.54	−0.41
	CM01	Vf_Mt5g015280_001	T:C	62.66	0.00499	4.18	0.37	−0.39
	CM02	**Vf_Mt3g086600_001**^g^	G:T	103.65	0.00001	8.84	0.89	−0.87
	CM02	Vf_Mt4g007030_001	C:A	234.93	0.30018	5.14	0.34	−0.43
	CM03	Vf_Mt1g105040_001	G:T	97.13	0.00733	4.23	0.85	−0.52
	CM03	Vf_Mt1g056180_001	A:G	227.32	0.00885	3.74	0.29	−0.38
	CM04	Vf_Mt2g086880_001	T:A	62.66	0.00289	4.98	0.40	−0.41
LCAF	CM01	**Vf_Mt5g046030_001**^g^	C:T	293.36	0.00043	8.20	0.53	−0.54
	CM01	Vf_Mt2g027240_001	C:T	401.92	0.00143	5.44	0.54	−0.44
	CM02	**Vf_Mt3g086600_001**	G:T	103.65	0.00009	7.93	0.89	−0.85
	CM02	Vf_Mt5g075540_001	G:C	31.06	0.00304	5.20	0.49	−0.43
	CM03	Vf_Mt1g105040_001	G:T	97.13	0.00733	4.95	0.85	−0.54
	CM04	Vf_Mt2g086880_001	T:A	62.66	0.00364	4.93	0.40	−0.42

Traits, AUSPC; area under symptoms progress curve after hardening conditions; LTAF, loss of leaf turgidty after frost; LCAF, loss of leaf color after frost.

a Linkage group, CM refers to the consensus map, LG refers to genetic linkage in BPP.

b “Left” allele decreases the trait.

c Position of the marker on the respective linkage group in CM or genetic linkage in BPP.

d Phenotypic variation explained by marker (%).

e The allele frequency was calculated for the allele which decreases the trait.

f The effect of the left allele.

g Marker showed significant association using MLM+kinsip at 20% FDR.

Bold markers showed significant association at 5% FDR.

### QTL for frost tolerance in both populations

In the RILs mapping population, two and three QTL were identified for M_AUSPC and H_AUSPC (Table 1, Figure 1), respectively. For H_AUSPC, two QTL, H_AUSPC_1, and H_AUSPC_2, were located in LG02 in the marker intervals 19A15_3-VF_MT5G026780_001 and VF_MT5G033880_001-VF_MT5G005120_001 with a peak *F*-value of 16.7 and 20.2%, respectively. H_AUSPC_3 was mapped on LG10 in marker interval VF_MT7G084010_001-VF_MT7G090890_001 with a peak of *F*-value 19.3. The PVE of H_AUSPC ranged from 13.15% (H_AUSPC_2) to 18.79% (H_AUSPC_1). The two QTL of M_AUSPC were located on LG02 and LG10 in intervals VF_MT1G016750_001-19A15_3 and VF_MT7G084010_001-VF_MT7G090890_001. The PVE of these QTL were, approximately the same, 14.35% for M_AUSPC_1 and 14.79% for M_AUSPC_2. All the five QTL showed a negative additive effect indicating that the alleles for decreasing AUSPC (symptoms of frost injury) were contributed by Côte d'Or 1. No QTL were found for U_AUSPC.

**Figure 2:**
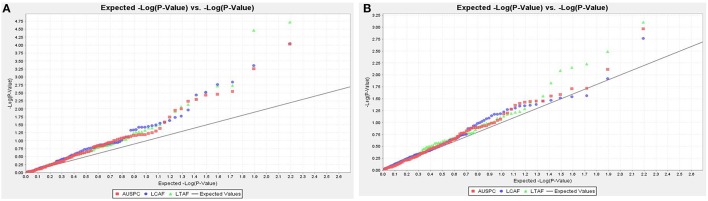
**The Q–Q plot shows the expected −log (P) vs. the −log (P) for AUSPC, LCAF, and LTAF. (A)** GLM **(B)** MLM + kinship.

Association mapping analysis was performed on GWBP using 156 SNP markers. The results revealed 11 QTL for AUSPC, eight for LTAF, and six for LCAF using GLM model at 20% FDR. Two significant markers of Vf_Mt5g046030_001 and Vf_Mt3g086600_001 were found to be associated with LCAF and LTAF using MLM+kinship at 20% FDR. Moreover, two significant markers Vf_Mt5g046030_001 and Vf_Mt3g086600_001 showed marker-trait association with the three frost tolerant traits using GLM at 0.05 FDR. The population structure and linkage disequilibrium of GWBP with the same set of 156 SNP markers were extensively described in Sallam and Martsch (2015) and Sallam et al. (2015a). The distribution of observed −log10 *P*-values for the three traits in both models is presented by quantile–quantile plot (Q–Q plot) in Figure 2.

### QTL for physiological traits associated with frost tolerance in BPP

The leaf FAC was analyzed in two treatments (hardening and unhardening) (Table 1). The proline content was analyzed only after hardening.

A total of three and nine QTL were found for FAC after unhardening (two fatty acids) and hardening treatments (six fatty acids), respectively. In hardening treatment, different QTL were found for most FAC contents. Three QTL associated with H_C18:1 and only one QTL for H_C16:0, H_C18:2, H_C18:3, and H_C18:4. Two major QTL were found with a PVE of 22.27% (H_C18:0_1) and 29.41% (H_C18:1_3). The lowest PVE among FAC and among all QTL detected in the present study was found for H_C18:1_1 with 2.74%. All QTL of FAC were distributed on LG02, LG03, LG04, LG08, and LG10. The highest number of QTL after hardening conditions were focused in LG02 and LG03 with three QTL each. The positive alleles for high content of C18:2 and C18:3 after hardening were contributed by Côte d'Or 1. The same parent contributed for low content of C16:0, C18:0, and C18:4 after hardening treatment. For C18:1 content after hardening, both parents contributed with a high content of C18:1. BPL4628 contributed with a high C18:1 at two marker intervals 19A15_3-VF_MT5G026780_001 and VF_MT4G113270_001-VF_MT4G104240_001, while, Côte d'Or 1 contributed with a high content of C18:1 at the VF_MT7G090890_001-VF_MT7G090930_001 marker interval with allele additive effects of 0.64.

In unhardening conditions, only two QTL were detected for U_C16:0 and one QTL for U_C16:1. LG03 including two QTL U_C16:0_2 and U_C16:1 in marker intervals of VF_MT4G127690_001-VF_MT4G125100_001 and VF_MT4G114900_001-VF_MT4G113270_001. U_C16:1 accounted for the highest PVE for with 14.55%, on the other hand, the lowest PVE was found for U_C16:0_2 with 11.28%. Côte d'Or 1 had positive and negative alleles for high (VF_MT3G077670_001-VF_MT3G086600_001) and low (VF_MT4G127690_001-VF_MT4G125100_001) contents of C16:0 and only positive alleles to increase C16:1 content at VF_MT4G114900_001-VF_MT4G113270_001 locus.

**Figure 3:**
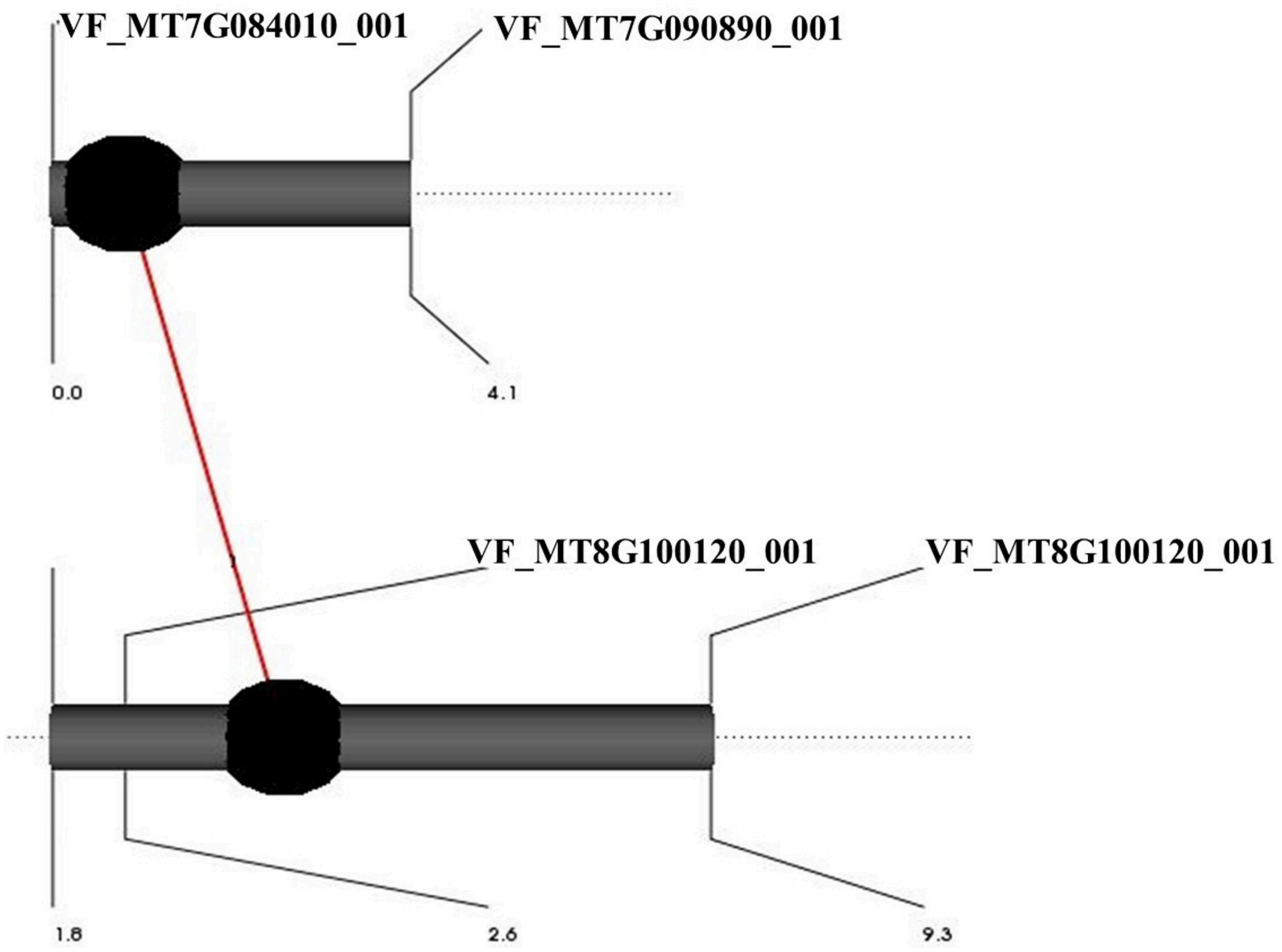
**Graphic representation of QTLs showing interaction effects for proline content in BPP (101 RILs)**. Black circles refer to QTLs with additive effects. The red line refers to significant interaction between the main effects of two QTLs.

An epistatic interaction between different QTL controlling the same trait was examined using a full-QTL model employing Markov Chain Monte Carlo algorithm of the QTL Network software. Among all traits, only free proline content showed an epistatic interaction (Table 2). QTL proline_1 located in marker interval VF_MT8G101390_001-VF_MT8G100120_00 on LG04 showed significant additive by additive interaction with proline_2 located in marker interval VF_MT7G084010_001-VF_MT7G090890_001 on LG10 (Figure 3). The epistatic interactions between both QTL contributed to 25.34% of the total phenotypic variance.

Remarkably, two marker intervals showed phenotypic effects that affect more than one QTL. The VF_MT7G084010_001-VF_MT7G090890_001 marker interval was found to be associated with two QTL associated with frost tolerance (H_AUSPC_3 and M_AUSPC_2) on LG10, while, three QTL associated with FAC (H_C18:0_2, H_C18:1_2, and H_C18:3) after hardening treatment were mapped in the same marker interval of VF_MT4G113270_001-VF_MT4G104240_001 on LG03.

### Genetic diversity analysis

Of the 189 SNP markers, 186 and 129 showed polymorphism in GWBP and BPP populations, respectively. PIC and gene diversity (GD) of the SNPs in both of the GWBP and BPP populations are illustrated in Figures S4A,B. The PIC of SNPs in GWBP and BPP populations averaged 0.28 and 0.37, with ranges of 0.05–0.38 and 0.25–0.38, respectively. The gene diversity of SNPs in GWBP and BPP populations averaged 0.36 and 0.49, with ranges of 0.05–0.50 and 0.30–0.50, respectively. Furthermore, a high significant correlation was found between PIC values of the common SNP markers (*N* = 129) (*r* = 0.80^**^) in both populations (Figure S4C). Likewise, GD values of the common markers in both population showed a high significant correlation (*r* = 0.94^**^).

By investigating the SNP markers showing high levels of PIC and gene diversity in GWBP and BPP populations, a common set of 54 SNPs were recorded highly polymorphic, for the first time, in both of the two different backgrounds populations of faba bean (GWBP and BPP) (Table S5). Of this promising set of 54 SNPs, 47 were mapped in the faba bean consensus map (Webb et al., 2015) and 7 were distributed across 5 linkage groups in the faba bean genetic map of the current study (Table S5).

### QTL validation and synteny analysis

QTL validation was tested by looking for significant markers associated with frost tolerance and related traits in GWBP and BPP populations. The results revealed that five SNP markers showed significant marker-trait association in both populations (Table 4). These markers were found to be associated with frost tolerance and its related traits in BPP and GWBP. Moreover, synteny between faba bean and *M. truncatula* genomes was investigated for these markers. QTL corresponding to *Medicago trucatula* genes are shown in Table 4 which also shows annotated gene information on positional candidate genes for the five markers.

**Table 4:** **Markers showing significant marker-trait association (FT, frost tolerance; WH, winter hardiness; and YA, yield attributes) in two different genetic backgrounds (BPP and GWBP) and their corresponding gene annotation**.

**Faba bean loci**	***M. truncatula loci***	**Chro**.	**QTL in BPP**	**Type^a^**	QTL in GWBP^*^	**Type^a^**	**Functional annotation of the gene**
VF_Mt5g026780	Medtr5g026780	5	H_AUSPC_1 H_C18:1_1	FT FAC	DS^b^, I_1_ (DS)^b^ FPHc, seed yieldc	FT YA	Neutral amino acid transporter
VF_Mt3g086600	Medtr3g086600	3	H_C16:0_1	FAC	DSb, FTIb, I_1_ (DS)b, I_2_ (REG)^b^, LT+LC^b^, RWCAF^b^, RDF^b^, AUSPC, LTAF, LCAF WRS^c^ Seed yield^c^	FT FT FT FT WH YA	Hypothetical protein
VF_Mt4g127690	Medtr4g127690	4	U_C16:0_2	FAC	DS^b^, I_1_ (DS)^b^ C16:0^a^, SFA^a^ DTF^c^	FT FAC YA	Transmembrane protein
VF_Mt4g125100	Medtr4g125100	4	U_C16:0_2	FAC	C16:0^b^	FAC	Cupin family protein
VF_Mt2g027240	Medtr2g027240	2	H_C18:4	FAC	DS^b^, I_1_ (DS)^b^, LT+LC^b^, LCAF LTAF, AUSPC SFA^b^	FT FT FAC	Serine/Threonine kinase family protein

a Type of QTL.

b Traits scored by Sallam and Martsch (2015).

c Traits scored by Sallam et al. (2015b).

* DS, disposition to survive after frost, I_1,_ index 1 (including DS); FPH, field plant height; FTI, FT index; LT+LC, loss of color + loss of turgidity; RDF, reduction in water content due to frost stress; RWCAF, relative water content after frost; I_2_, index 2 (including regrowth after frost); SFA, saturated fatty acid content; DTF, days to flowering.

**Figure 4:**
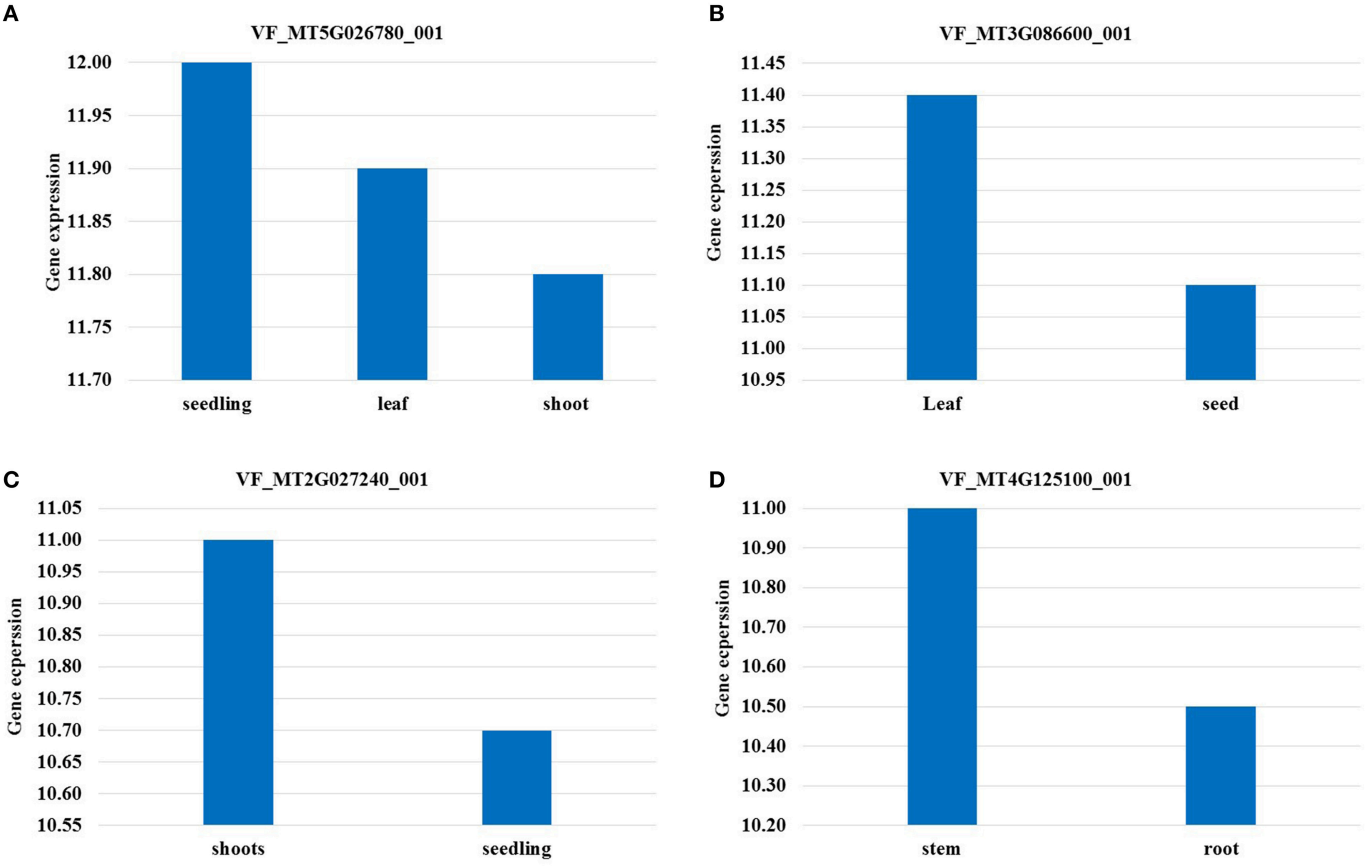
**Highest expression of candidate genes in different tissues**. The gene expression was considered in tissue which was not exposed to any treatment (under control conditions).

VF_Mt5g026780 was found to be associated with frost tolerance in both populations. The candidate gene for this marker encodes a neutral amino acid transporter. The expression of this gene was found to be highest in seedling stage followed by leaves in *M. truncatula* (Figure 4A). Two markers VF_MT4G125100_001 and VF_MT4G127690_001 were found to be associated with C16:0 content in both populations. The candidate genes of these markers encode to cupin family protein and transmembrane protein, respectively. VF_Mt3g086600 was found to be associated with H_C16:0_1 in BPP, and with ten frost tolerant traits in GWBP. The candidate gene of VF_Mt3g086600 encode a hypothetical protein which was highly expressed in leaves of *M. truncatula* (Figure 4B). A segment of *M. truncatula* that harbors a Serine/Threonine kinase family protein was associated with H_C18:4, frost tolerance and saturated fatty acid in GWBP. It showed a high gene expression in *M. truncatula'*s shoot (Figure 4C). Two SNP markers VF_Mt4g125100 and VF_; Mt4g127690, were located on chromosome 4 of *M. truncatula,* while, only one SNP marker was located on chromosomes 2, 3, and 5 of *M. truncatula.*

## Discussion

### Genetic variation in frost tolerance and related traits in both populations

The high genetic variation between genotypes in BPP and GWBP for all traits promises the improvement of frost tolerance in winter faba bean in breeding and genetics programs. Moreover, the high repeatability estimates make selection for high frost tolerance both possible and fruitful. The AUSPC showed approximately the same repeatability estimates in BPP (*h*^2^ = 0.89, Arbaoui et al., 2008b) and in GWBP (*h*^2^ = 0.90). Both populations include different degrees of frost tolerance that can be used to improve winter faba bean, however, GWBP may include a wider range of frost tolerance than BPP due to the nature by which this population was produced. The GWBP population was derived from crossing between 11 different parents. The genetic distance between the 11 parents of both populations was studied by Sallam and Martsch (2015). The common parent between BPP and GWBP is Côte d'Or/1 (frost tolerant) which contains favorable alleles for frost tolerance (Arbaoui et al., 2008b; Sallam and Martsch, 2015). The physiological traits showed significant but low correlations with frost tolerance in BPP. The same findings were reported by Sallam et al. (2015b). FAC and proline content were reported as useful indicators to frost stress tolerance in faba bean and cereals (Bates et al., 1973; Stoddard et al., 2006). Sallam et al. (2015b) reported that studying FAC after frost may also be informative due to its strong correlations with frost tolerance in faba bean.

### Genetic linkage map

In the present study, a molecular genetic linkage map of winter faba bean was constructed using 117 SNP markers to identify QTL associated with frost tolerance and related traits. The average interval was 3.21 cM (Table S3). In genome wide QTL scanning, an average interval of less than 10 cM is recommended as a marker density (Doerge, 2002). Thus, the faba bean genetic map constructed in this study was quite appropriate for mapping QTL for target traits. Obviously, some gaps were found in the genetic map with the longest gap of 10 cM on LG01. These gaps may indicate failure to detect QTL in such genomic regions (Kumawat et al., 2012). Large gaps were also found in FBCM_03 (Figure S2, Webb et al., 2015). The number of faba bean chromosomes has been reported as 2*n* = 12. Some earlier studies also reported genetic maps with 13 (Patto et al., 1999) and 18 (Avila et al., 2004) linkage groups. Khazaei et al. (2014) constructed a faba bean genetic map (nine linkage groups) using 188 SNP markers which also were mapped in FBCM. Arbaoui et al. (2008b) constructed a faba bean genetic linkage map with 21 linkage groups using RAPD markers to map QTL associated with frost tolerance in the same population used in this study (101 RILs). This difference between the number of linkage groups and the number of faba bean chromosomes may be due to the large genome (~13,000 Mb) of faba bean compared to other legumes (Ellwood et al., 2008).

Interestingly, a set of 105 SNP markers were mapped in FBCM. Thus, a direct comparison between the two maps was possible. The 14 linkage groups of the current genetic map were randomly attached to the six linkage groups of FBCM, covering many genomic regions in FBCM. Each linkage group in FBCM was assumed to correspond to one chromosome of the six *V. faba* chromosomes (Webb et al., 2015). Homology between faba bean genome and other legume species genomes was previously reported (Ellwood et al., 2008; Satovic et al., 2013; Webb et al., 2015). The order of SNP markers in the linkage groups of the current genetic map of faba bean matched the order of the same SNP markers in FBCM.

### QTL associated with frost tolerance

The same 101 RILs of BPP was previously used to detect QTL associated with frost tolerance using RAPD markers (Arbaoui et al., 2008b). We re-genotyped the same lines and their parents using 189 SNP markers and performed QTL analysis using the same traits. Unlike all other DNA molecular markers, SNPs can be utilized to develop haplotyping systems for genes or regions of interest (Rafalski, 2002). Furthermore, SNPs are useful when several define haplotypes in the region of interest. Another reason for genotyping the same lines using SNPs was that this set of 189 markers was previously used to genotype GWBP. Therefore, validation of QTL associated with frost tolerance and identification of the candidate genes controlling frost tolerance in winter faba bean could be possibly investigated.

A range of 100–500 individuals is recommended for association and QTL mapping studies. Furthermore, the use of co-dominant SSRs and SNPs is more informative (no allelic ambiguity) than other dominant markers (Kumar et al., 2011). Thus, the numbers of genotypes of BPP (101) and GWBP (189) were quite suitable for detecting QTL controlling frost tolerance and its related traits. Unfortunately, there are very few studies reporting QTL for frost tolerance in winter faba bean (Arbaoui et al., 2008b; Sallam and Martsch, 2015).

A total of 17 QTL were identified covering two frost tolerant traits and eight physiological traits. This number of QTL was higher than those reported by Arbaoui et al. (2008b) using RAPD markers (12 QTL). In a bi-parental mapping population, the number of traits with loci having contrasting alleles between two parents affects the number of identified QTL (Mackay and Powell, 2007). In current BPP, although Côte d'Or/1 and BPL4628 are frost tolerant winter faba bean genotypes, they are genetically divergent. Sallam and Martsch (2016) found that the genetic distance between the two parents, using 189 SNP markers, was 0.964. This explains the high number of QTL indentified in the present stduy. Of these 17 QTL, 11 have major effects with a PVE of higher than 14%. Frost tolerance of winter faba bean is a very important trait which is controlled by many additive genes (Duc and Petitjean, 1995). Therefore, faba bean can be genetically improved using plant breeding programs along with MAS. Côte d'Or/1 alleles at all the five loci decreased the symptoms of frost stress on winter faba bean seedlings. The H_AUSPC_3 allele showed the maximum decrease of frost stress symptoms with an effect of 30.38.

In GWBP, the association mapping was conducted on the different genetic background of 189 SSD lines derived from a natural crossing between 11 parents (Côte d'Or/1 is one of them). The linkage disequilibrium and population structure of GWBP were described in detail by Sallam and Martsch (2015). Because Sallam and Martsch (2015) and Sallam et al. (2015a) confirmed that GWBP has low structure, we assume that GLM model fits with the analysis of association mapping. However, we presented marker-trait association using MLM+K for AUPSC, LTAF, and LTCF. The results of association analysis revealed that 12 different significant markers were found to be associated with AUSPC, LTAF, LCAF (Table 3). It was difficult to determine the contribution of each parent of the GWBP (unlike BPP) because this population was derived from a natural crossing of 11 parents. However, the number of alleles (for the 12 significant markers, Table 3) associated with decreased frost stress symptoms in each founder line is presented in Figure S3A. This number ranged from four alleles inherited from Banner/1 to ten alleles from L979/1. This indicates that L979/1 is the main source of frost tolerance found in GWBP. Of 12 alleles, Côte d'Or/1 had eight alleles that were responsible for decreasing the effect of frost stress. Conversely, for each allele associated with a decrease in frost stress symptoms, the number of founder lines carrying that allele was determined (Figure S3B). This was done in order to show the prevalence of each allele among founder lines. For example, two alleles A (Vf_Mt8g040550_001) and T (Vf_Mt5g046030_001) were inherited from one and/or two of the nine possible founder lines. The frost tolerance index (FTI) for the GWBP and their 11 parent was reported by Sallam (2014). Unlike with the QTL mapping of BPP, a genome wide association study allows us to identify alleles and loci responsible for important traits associated with stress tolerance and agronomic features (Kraakman et al., 2004). Côte d'Or/1 is a very important source for frost tolerance alleles in GWBP and BPP. The PVE of QTL in GWBP was very low (range of 2.27–7.93%) compared to those detected in BPP (2.74–25.34%).

### QTL for physiological traits associated with frost tolerance in BPP

Putative QTL were detected for FAC and proline content. These physiological parameters are potentially useful indicators of the frost stress in cereals and legumes (Maqbool et al., 2009; Link et al., 2010). Among physiological traits, the highest number of QTL detected in BPP was found for FAC. Most of the putative QTL for FAC were detected in LG03 which was colinear with CM06 in FBCM. After hardening, Côte d'Or/1 possessed alleles which were associated with decreased C16:0 and C18:4 contents, and increased C18:2 and C18:3. Moreover, Côte d'Or/1 had alleles associated with increased and decreased C18:1. In the membrane lipids of chilling-resistant plants, Williams et al. (1988) and Palta et al. (1993) found a high proportion of unsaturated fatty acid (C18:2 and C18:3) and a low portion of saturated fatty acid (C16:0 and C18:0) during acclimation to cool temperatures. Unsaturated fatty acids help the faba bean leaves to be more fluid during frost stress. Three QTL controlling FAC were located in the marker interval of VF_MT4G113270_001-VF_MT4G104240_001 which showed pleiotropic effects. This locus was found to be associated with decreased C18:0 and C18:1, and increased C18:3 under hardening conditions. This is in agreement with the correlation pattern between traits. After hardening, C18:3 was found to be significantly correlated with C18:1 (*r* = −0.56^**^, Arbaoiu et al., unpublished data). Sallam and Martsch (2015) detected one QTL for saturated fatty acid (SFA—C16:0 and C18:0 contents), eight QTL for C16:0 content, and nine QTL for unsaturated fatty acid content after hardening conditions in GWBP. Arbaoui et al. (2008b) detected only three QTL controlling C18:1 and C18:2 contents after hardening in the same population (101 lines) using RAPD markers. The source of positive alleles was Côte d'Or/1. Epistatic interaction between Proline_1 and Proline_2 for proline content was significant, accounting for 25.34% of the phenotypic variation. This indicates the need for taking into account both additive and epistatic effects for devising an effective molecular breeding strategy (Kumawat et al., 2012). The correlation between proline accumulation during hardening and frost tolerance induction was previously reported in many species, e.g., wheat (Dörffling et al., 1990) and faba bean (Arbaoui et al., 2008b).

Côte d'Or/1 has several alleles which decrease the symptoms of frost (in BPP and GWBP) and saturated fatty acid content. Moreover, it has alleles increasing unsaturated fatty acid content and proline which are in favor of frost tolerance gain. These traits, especially AUSPC can be utilized in winter faba bean improvement using marker assisted breeding. It was also concluded that Côte d'Or/1 is the main source of frost tolerance in BPP and one of the main contributors for frost tolerance in GWBP.

**Figure 5:**
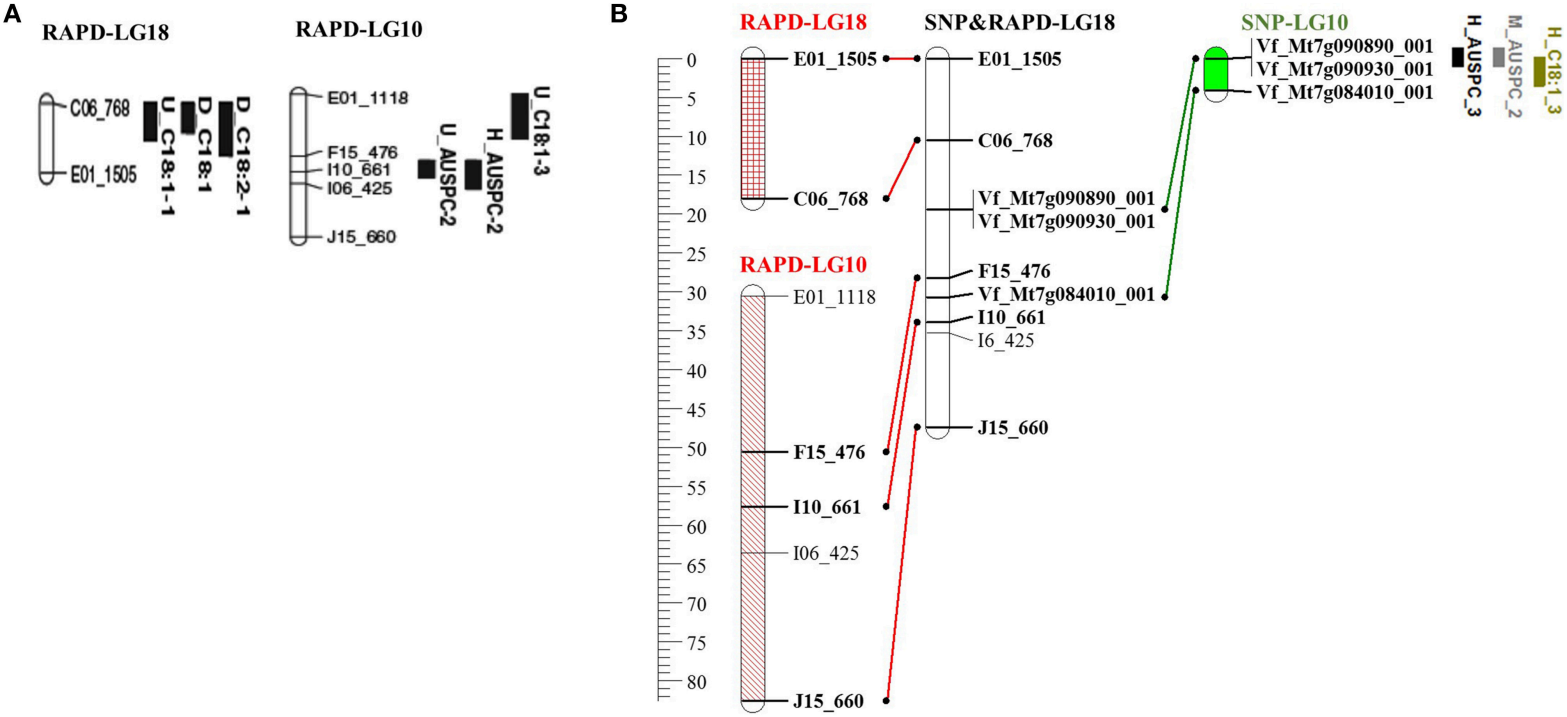
**(A)** Linkage groups associated with putative QTL for frost tolerance (Arbaoui et al., 2008b), **(B)** The collinearity between SNP&RAPD-LG18 constructed by Sallam et al. (unpublished data, doi: 10.6084/m9.figshare.3471665, https://figshare.com/s/3aee3d0182a75c7375a9), SNP-LG10 of the genetic map was constructed in the present study, and both RAPD-LG18 and RAPD-LG10 were constructed by Arbaoui et al. (2008b). Markers on SNP-LG10 are located on chromosome 7 of *M. truncatula* and collinear with FBCM_05.

As mentioned above, BPP (101 RILs) was previously used to construct genetic linkage map using 131 RAPD markers (Arbaoui et al., 2008b). We combined the RAPD from Arbaoui et al. (2008b) and the SNP markers used in this study to construct a combined genetic linkage map using both types of markers (Sallam et al., unpublished data, doi: 10.6084/m9.figshare.3471665, https://figshare.com/s/3aee3d0182a75c7375a9). Out of 131 RAPD markers, 112 were mapped along with 112 SNP markers in the combined genetic linkage map. The positions of QTL detected by SNP markers (in the current study) were compared with the positions of those RAPD markers associated with QTL detected by Arbaoui et al. (2008b). These results revealed that the markers in linkage group 10 in the current faba bean genetic map (SNP markers), associated with three QTL (H_AUSPC_3, M_AUSPC_1, and H_C18:1_3), were mapped with the RAPD-marker intervals of C0_768—E01_1505 (RAPD-LG18) and F15_476—I10_661 (RAPD-LG10) in the combined SNP&RAPD genetic linkage map (SNP&RAPD-LG18, Figure 5). The first RAPD-marker interval C0_768—E01_1505 was found to be associated with also three QTL (U_C18:1-1, D_C18:1, and D_C18:2-1), while, the second RAPD-marker interval F15_476—I10_661 was associated with U_AUSPC-2 and H_AUSPC-2 (Arbaoui et al., 2008b). All these marker intervals were mapped together in the combined RAPD&SNP-LG18 in the genetic linkage map constructed using both types of markers. The SNP-marker interval of VF_MT7G090890_001-VF_MT7G090930_001 was located between the two RAPD-marker intervals. Moreover, the VF_MT7G084010_001 was mapped in the RAPD-marker interval F15_476-I10_661. This indicates that this genomic region may include very important genes controlling frost tolerance since the RAPD-marker interval (F15_476-I10_661) and the SNP marker (VF_MT7G084010_001) were associated with AUPSC. These marker intervals located on RAPD&SNP-LG18 were found to be associated with frost tolerance and FAC (C18:1 and C18:2). It was SNP interval VF_MT7g090890_001-VF_MT7g090930_001 that made it possible to connect the two RAPD linkage groups, RAPD-LG18 and RAPD-LG10, to make one linkage group, SNP&RAPD-LG18, in the combined genetic map. Using collinearity of SNP markers, this linkage group was attached with FBCM_05 (Figure S2) and chromosome 7 in *Medicago truncatula*. This illustrates another benefit of the collinearity study, showing possible positions of interesting genomic regions controlling target traits. Moreover, combining many types of markers to construct a genetic map could be useful since each type of marker demonstrates different advantages in detecting genomic regions.

### Genetic diversity analysis

Based on the SNP markers genotyped in the current study, the average values of PIC and gene diversity in BPP and GWBP populations were higher than that were reported by Suresh et al. (2013) and Kaur et al. (2014) on faba bean populations, indicating that these markers could be very efficient for assessing the genetic variability and population structure of faba bean. In light of novel observations, a common set of 54 SNPs were recorded as highly polymorphic in both of the two different genetic background populations (GWBP and BPP) (Table S5). This original set of 54 SNP markers has proven to be more efficient than the whole set of SNPs in assessing the genetic diversity of the two different genetic backgrounds of faba bean, and could be exploited, for the first time, to genotype many faba bean populations regardless of their genetic backgrounds. This novel set of SNPs could help in QTL mapping and marker-assisted selection (MAS) studies, and consequently has a great impact on accelerating faba bean breeding practices for crop improvement. Additionally, due to the high distribution throughout the whole genome of faba bean, this set of SNPs is highly recommended as the anchor set of faba bean SNPs for the present and future applications in large-scaled genetic studies of faba bean. Fortunately, due to the high density of SNPs in the faba bean genome and availability of the promising sequencing platforms, it is possible to proceed in the broadening of this anchor set of SNPs in order to develop multiple SNP sets of different throughputs which will benefit all faba bean breeders worldwide.

### QTL validation and synteny analysis

Validation of QTL controlling target traits is an essential step in MAS. The QTL validation in this study was performed in a different genetic background. The same SNP markers (156 SNPs) were used before for genome wide association study to detect alleles associated with frost tolerance, winter hardiness, and yield attributes in GWBP (Sallam and Martsch, 2015; Sallam et al., 2015a). Five SNP markers showed marker-trait association in both different genetic backgrounds (BPP and GWBP). VF_MT5G026780_001 marker was associated with frost tolerance (H_AUSPC_1) in BPP and in GWBP (DS, disposition to survive after frost). This segment on chromosome 5 of *M. truncatula* encodes a neutral amino acid transporter which is included in all processes that are associated with the allocation of nitrogen during plant growth (Chen, 2001). The candidate gene of the neutral amino acid transporter showed high expression in seedling stage followed by leaves of *M. truncatula*. All traits associated with this SNP marker were scored on faba bean leaves at seedling stage.

The candidate gene of VF_MT3G086600_001 has markers on chromosome 3 on *M. truncatula*, which was found to be associated with H_C16:0_1 in BPP and with the highest number of QTL controlling frost tolerance and related traits in GWBP, encodes a hypothetical protein which is defined as “a protein with information available for its translation.” This protein is normally expressed from an open reading frame (Desler et al., 2009). This candidate gene was highly expressed in *M. truncatula* leaves (Figure 4). VF_MT3G086600_001 was found to be associated with leaf C16:0 content after hardening in BPP and with loss of leaf turgidity +loss of leaf color (LT+LC), reduction in leaf water content due to frost stress (RDF), relative water content after frost in leaves (RWCAF), LTAF, LCAF, and H_AUSPC. All of these traits were scored in faba bean leaves.

Interestingly, two segments on chromosome 4 were found to be associated with C16:0 content in both populations. These segments harbor a transmembrane protein and a cupin family protein. An increase in transmembrane proteins was found by the covalent attachment of a fatty acid chain (Alberts et al., 2002). No information is available about the relation between cupin family protein and fatty acid in plants.

The candidate gene of Mt2g027240, which associated with H_C18:4 in BPP, with frost tolerance and FAC in GWBP, encodes to a serine/threonine kinase family protein which plays a critical role in the regulation of lipid metabolic activity and controls the dyanamics of plant growth and regulation in *Arabidopsis thaliana* (Parthibane et al., 2012). More importantly, it was reported that serine/threonine kinase family proteins are involved with cold, salt stress, and seed development in peanut (Rudrabhatla and Rajasekharan, 2002) and with leaf senescence in soybean (Xu et al., 2011).

Bear in mind that the faba bean seedlings in both populations were exposed to different freezing temperature regimes. This explains why most of the significant markers might not be associated with the same trait in both populations but, this could be also an advantage of showing markers associated with frost tolerance under different degrees of freezing temperatures or/and hardening period (for FAC).

The high repeatability found for all traits in both populations indicates the effectiveness of the identification and validation of QTL performed in this study. It is very important to control the experimental errors in order to enhance broad-sense repeatability especially for QTL mapping with a large population (Wang et al., 2014).

*Medicago truncatula* is an interesting legume model for researchers due to its small genome (*M. truncatula* around 500 Mb, Gnanasambandam et al., 2012), which is much better suited to genetic and genomic research than large genomes such as that of *Vicia faba.* A simple and direct relationship was found between the two genomes of *M. truncatula* and *V. faba* (Ellwood et al., 2008). Therefore, the *M. truncatula* could be a good model for identifying genes controlling target traits (e.g., frost tolerance) in grain legumes.

In conclusion, the two different genetic backgrounds presented remarkable phenotypic variations in frost tolerance, which can be exploited in the dissection of the genetic architecture of frost tolerance. Based on the synteny between faba bean and *M. truncatula*, we found genomic regions on chromosome 4 (Table 4) and 7 (Figure 5) which showed association with frost tolerance and FAC which could be an interest for further studies. The comparison of new putative and validated QTL regions in faba bean with their syntenic regions in *M. truncatula* shed light on several valuable outcomes, such as possible genomic regions, which may control frost tolerance and its related traits. The candidate genes identified in this study may play important roles in improving frost tolerance in winter faba bean, however, further investigations are required on their biological function especially VF_MT3G086600_001which associated with frost tolerance, winter hardiness, and yield in winter faba bean.

## Author contributions

AS designed the study, performed all the statistical analyses, helped in phenotyping and genotyping of GWBP, and wrote the whole manuscript. MA phenotyped and genotyped the BPP. ME performed the analysis of genetic diversity, wrote the results and discussion of the genetic diversity analysis, and revised the manuscript. NA, helped in the discussion and drafted the manuscript. RM designed the experiments in Frost Growth Chamber, and helped in phenotyping and genotyping of GWBP and BPP.

### Conflict of interest statement

The authors declare that the research was conducted in the absence of any commercial or financial relationships that could be construed as a potential conflict of interest.

## Supplementary material

Figure S1 **Phenotypic correlation among frost tolerant traits scored on GWBP (189 lines)**.

Figure S2 **Collinearity between faba bean genetic linkage map constructed using 101 lines and faba bean consensus map (FBCM, Webb et al., 2015)**.

Figure S3 **(A) Number of alleles associated with decreased frost stress symptoms in founder lines (GWAS study), (B) number of founder lines carrying alleles associated with decreased frost stress symptoms**. Red column in **(A)** refers that this parent in not among GWBP's parents. ^*^Refers that CôteD‘Or/1 carries this allele.

Figure S4 **The distribution of polymorphic information content (A) and gene diversity (B) both populations (GWBP and BPP). Panel (C) refers to the correlation between PIC and gene diversity of the common SNP markers (***N*** = 126) in both populations**.

Table S1 **Minimum, maximum, mean, analysis of variation (F-values), and repeatability estimates for traits scored on BPP and GWBP**.

Table S2 **Phenotypic correlation between frost tolerance and physiological traits scored in BPP (101 RILs)**.

Table S3 **The properties of genetic linkage map of faba bean based on the BPP of Co$\hat{\text{t}}$e d'Or 1 (French landrace) and BPL 4628 (RILs mapping)**.

Table S4 **Localization of linkage groups of the current genetic map in the faba bean consensus map**.

Table S5 **List of 54 SNP markers showing a promising genetic diversity with high polymorphic information content (PIC) and gene diversity (GD) values in the two different genetic backgrounds (GWBP and BPP)**.

Table S6 **The sequences of the SNP markers used in GWAS and QTL mapping**.

Table S7 **SNP marker and their genetic distance in each linkage group in the current genetic map of winter faba bean (SNP markers)**.

Appendix 1 **List of traits abbreviations mentioned in the current study**.
