# Supplementary material for: Identification and Verification of QTL Associated with Frost Tolerance Using Linkage Mapping and GWAS in Winter Faba Bean
Source: Front Plant Sci. 2016 Aug 4;7:1098. doi: 10.3389/fpls.2016.01098 (PMC4972839; doi:10.3389/fpls.2016.01098)
Supplement: Figure S1 — Phenotypic correlation among frost tolerant traits scored on GWBP (189 lines). [file Presentation1.pptx]

## Slide 1
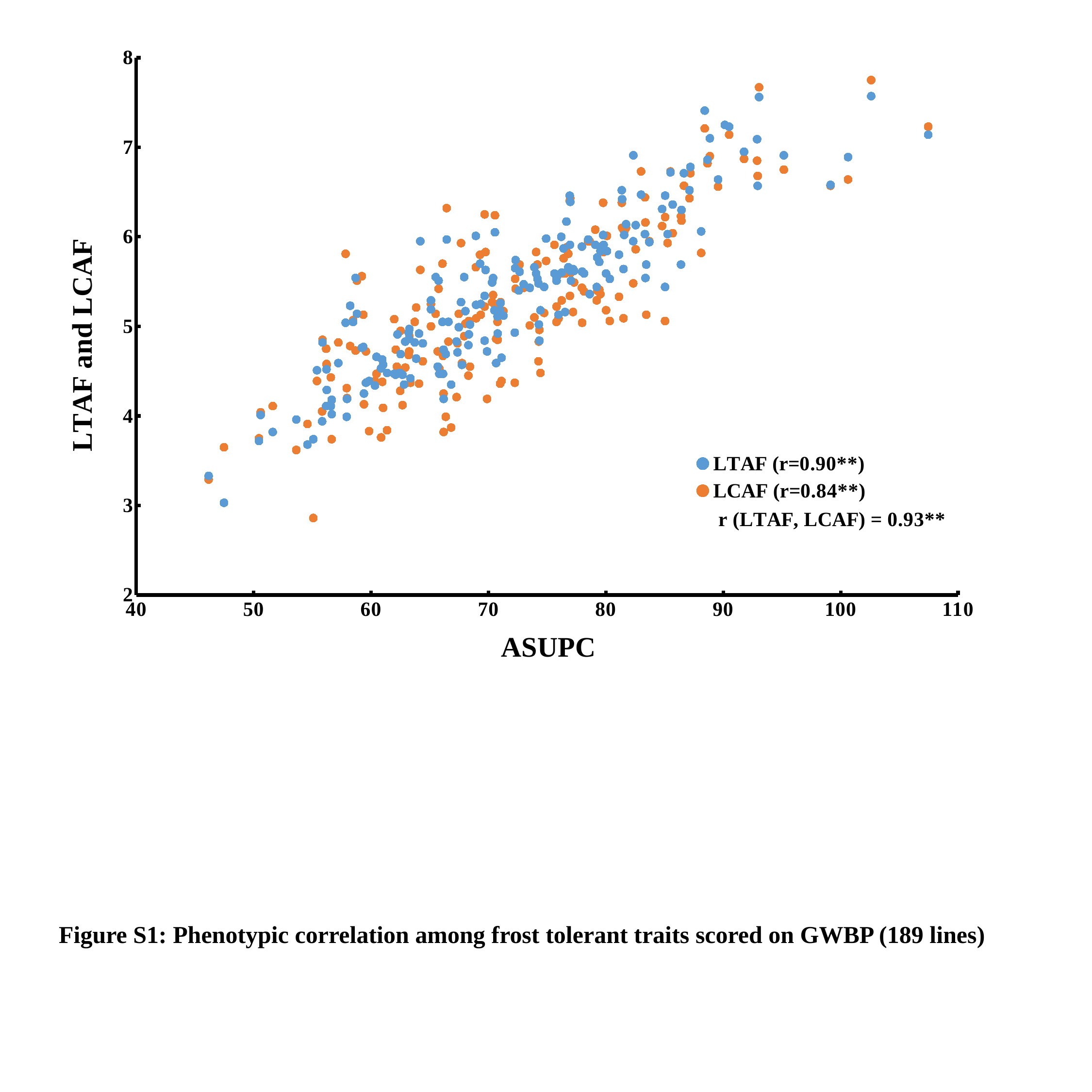

### Chart
| Category | | |
|---|---|---|Figure S1: Phenotypic correlation among frost tolerant traits scored on GWBP (189 lines)

## Slide 2
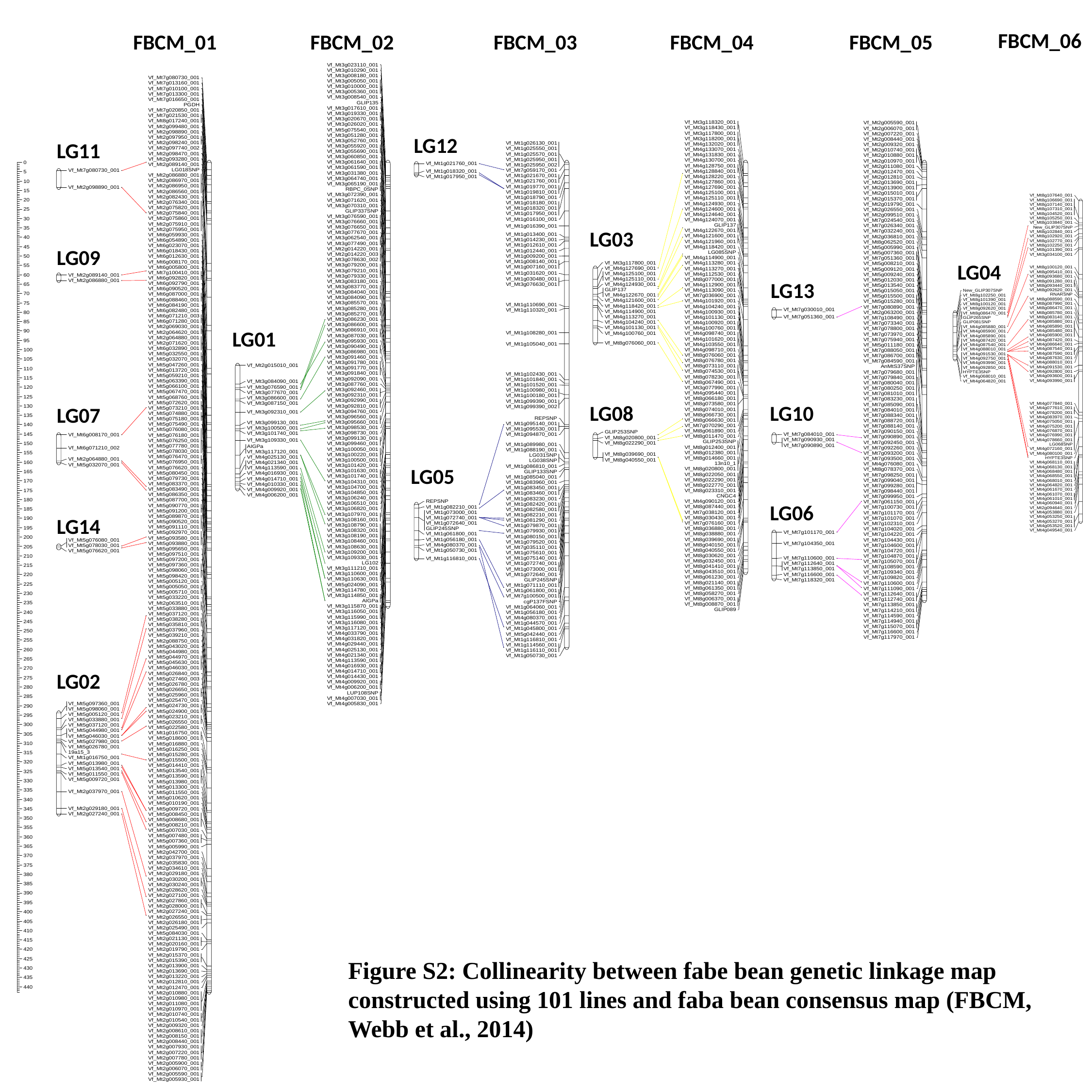

FBCM_06
FBCM_02
FBCM_01
FBCM_03
FBCM_04
FBCM_05
LG12
LG11
LG03
LG09
LG04
LG13
LG01
LG08
LG10
LG07
LG05
LG06
LG14
LG02
Figure S2: Collinearity between fabe bean genetic linkage map constructed using 101 lines and faba bean consensus map (FBCM, Webb et al., 2014)

## Slide 3
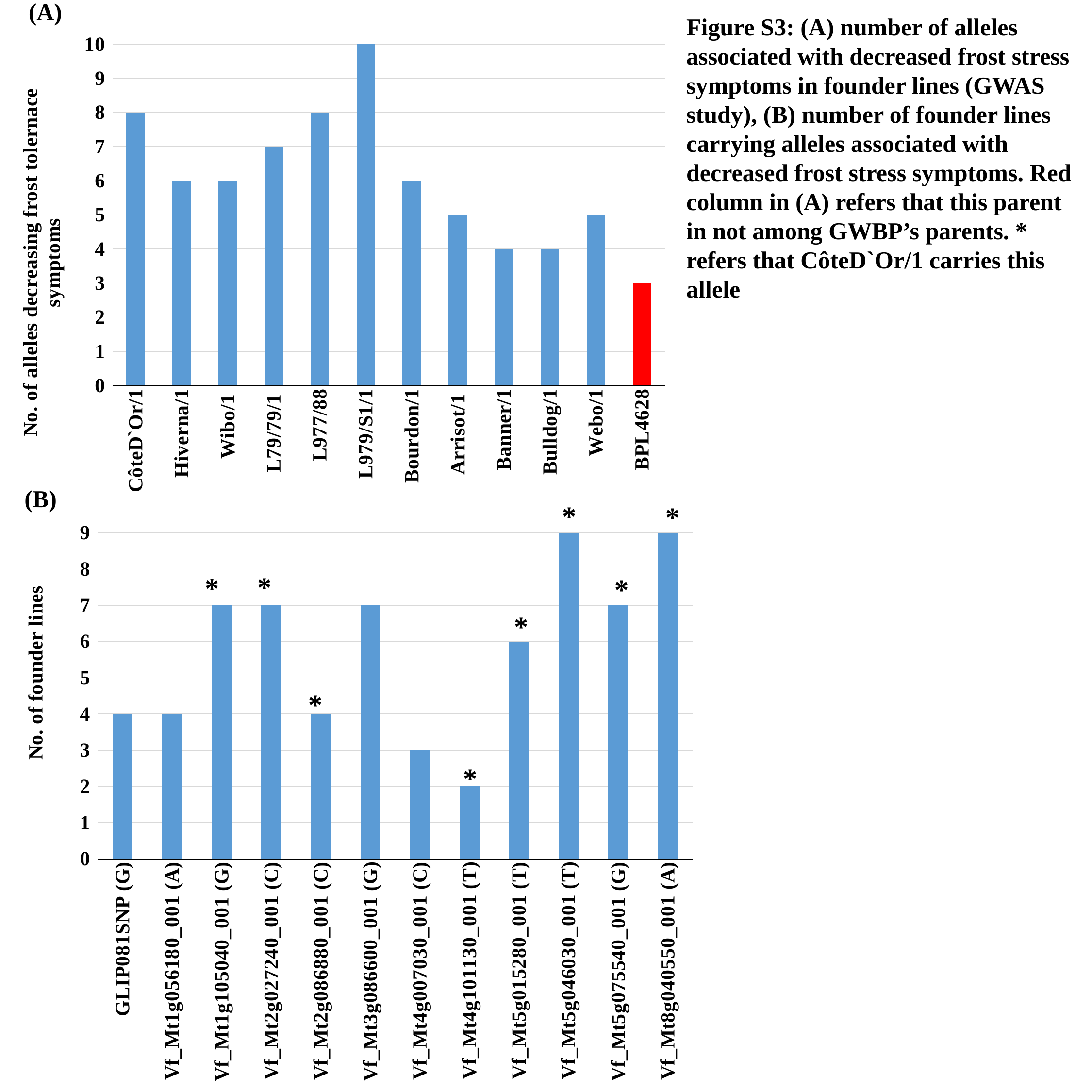

(A)
Figure S3: (A) number of alleles associated with decreased frost stress symptoms in founder lines (GWAS study), (B) number of founder lines carrying alleles associated with decreased frost stress symptoms. Red column in (A) refers that this parent in not among GWBP’s parents. * refers that CôteD`Or/1 carries this allele
### Chart
| Category | |
|---|---|
| CôteD`Or/1 | 8.0 |
| Hiverna/1 | 6.0 |
| Wibo/1 | 6.0 |
| L79/79/1 | 7.0 |
| L977/88 | 8.0 |
| L979/S1/1 | 10.0 |
| Bourdon/1 | 6.0 |
| Arrisot/1 | 5.0 |
| Banner/1 | 4.0 |
| Bulldog/1 | 4.0 |
| Webo/1 | 5.0 |
| BPL4628 | 3.0 |(B)
*
*
### Chart
| Category | |
|---|---|
| GLIP081SNP (G) | 4.0 |
| Vf_Mt1g056180_001 (A) | 4.0 |
| Vf_Mt1g105040_001 (G) | 7.0 |
| Vf_Mt2g027240_001 (C) | 7.0 |
| Vf_Mt2g086880_001 (C) | 4.0 |
| Vf_Mt3g086600_001 (G) | 7.0 |
| Vf_Mt4g007030_001 (C) | 3.0 |
| Vf_Mt4g101130_001 (T) | 2.0 |
| Vf_Mt5g015280_001 (T) | 6.0 |
| Vf_Mt5g046030_001 (T) | 9.0 |
| Vf_Mt5g075540_001 (G) | 7.0 |
| Vf_Mt8g040550_001 (A) | 9.0 |*
*
*
*
*
*

## Slide 4
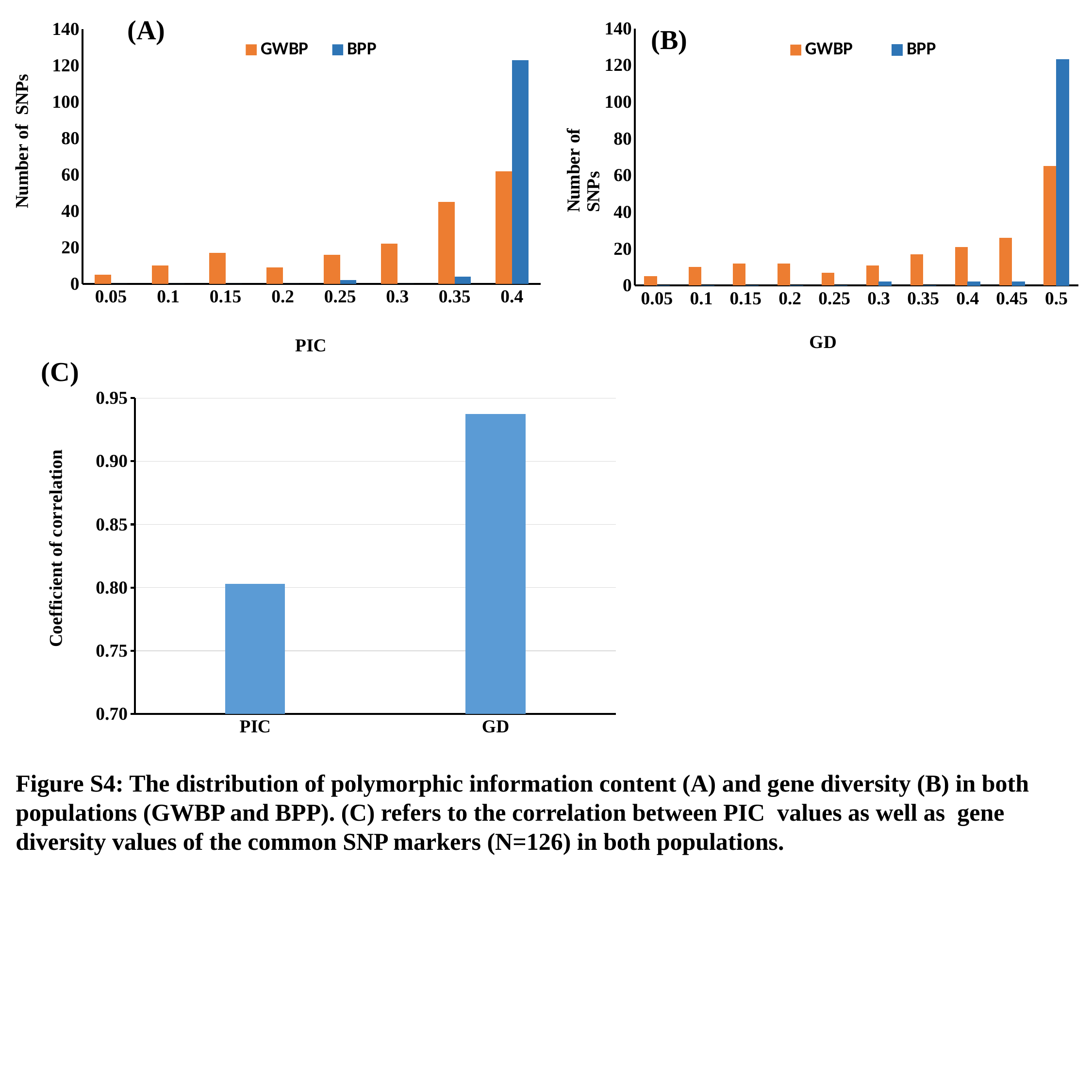

(A)
### Chart: PIC
| Category | | |
|---|---|---|
| 0.05 | 5.0 | 0.0 |
| 0.1 | 10.0 | 0.0 |
| 0.15000000000000002 | 17.0 | 0.0 |
| 0.2 | 9.0 | 0.0 |
| 0.25 | 16.0 | 2.0 |
| 0.30000000000000004 | 22.0 | 0.0 |
| 0.35000000000000003 | 45.0 | 4.0 |
| 0.4 | 62.0 | 123.0 |
### Chart: GD
| Category | | |
|---|---|---|
| 0.05 | 5.0 | 0.0 |
| 0.1 | 10.0 | 0.0 |
| 0.15000000000000002 | 12.0 | 0.0 |
| 0.2 | 12.0 | 0.0 |
| 0.25 | 7.0 | 0.0 |
| 0.30000000000000004 | 11.0 | 2.0 |
| 0.35000000000000003 | 17.0 | 0.0 |
| 0.4 | 21.0 | 2.0 |
| 0.45 | 26.0 | 2.0 |
| 0.5 | 65.0 | 123.0 |(B)
(C)
### Chart
| Category | |
|---|---|
| PIC | 0.8028432181260213 |
| GD | 0.9374525119857903 |Figure S4: The distribution of polymorphic information content (A) and gene diversity (B) in both populations (GWBP and BPP). (C) refers to the correlation between PIC values as well as gene diversity values of the common SNP markers (N=126) in both populations.
