# Supplementary material for: Identification and Verification of QTL Associated with Frost Tolerance Using Linkage Mapping and GWAS in Winter Faba Bean
Source: Front Plant Sci. 2016 Aug 4;7:1098. doi: 10.3389/fpls.2016.01098 (PMC4972839; doi:10.3389/fpls.2016.01098)
Supplement: Table S1 — Minimum, maximum, mean, analysis of variation (F-values), and repeatability estimates for traits scored on BPP and GWBP. [file Table1.docx]

Table S1. Minimum, maximum, mean, analysis of variation (F-values), and repeatability estimates for traits scored on BPP and GWBP.

| Trait | Minimum | | Maximum | | mean | F-value^(2)^ | h^2^ |
| --- | --- | --- | --- | --- | --- | --- | --- |
| Biparental population (BPP, 101 RILs) ^(1)^ | | | | |  |  |  |
| Proline | 24.00 | | 94.70 | | 52.60 | 82.70** | 0.98 |
| H_C16:1 | 1.23 | 2.82 | | 1.80 | | 1.93** | 0.75 |
| U_C16:1 | 1.24 | 2.12 | | 1.63 | | 2.27** | 0.65 |
| Gottingen Winter Bean Population (GWBP, 189 SSD lines) | | | |  | |  |  |
| AUSPC | 46.16 | 144.46 | | 72.75 | | 10.15** | 0.90 |
| LTAF | 2.07 | 8.89 | | 5.43 | | 15.49** | 0.94 |
| LCAF | 2.25 | 8.86 | | 5.535 | | 16.24** | 0.94 |

^(1)^ for fatty acid composition and frost tolerance (AUSPC) see Arbaoui et al. (2008a).

^(2)^ F-value of genetoypes.

** significant at the 0.01 level of the probability.

Table S2. Phenotypic correlation between frost tolerance and physiological traits scored in BPP (101 RILs)

| Trait | H_AUSPC | U_AUSPC | M_AUSPC |
| --- | --- | --- | --- |
| Proline | -0.28** | -0.17 | -0.25* |
| H_C16:0 | -0.12 | -0.21* | -0.18 |
| H_C16:1 | 0.16 | 0.19 | 0.19 |
| H_C18:0 | 0.24* | 0.12 | -0.12 |
| H_C18:1 | 0.12 | 0.35** | 0.30** |
| H_C18:2 | -0.14 | -0.28** | -0.25** |
| H_C18:3 | 0.02 | 0.04 | 0.02 |
| H_C18:4 | -0.02 | -0.05 | -0.02 |
| U_C16:0 | -0.04 | -0.12 | -0.10 |
| U_C16:1 | -0.01 | -0.03 | -0.02 |
| U_C18:0 | 0.20* | 0.22* | 0.24* |
| U_C18:1 | 0.10 | 0.16 | 0.17 |
| U_C18:2 | 0.04 | 0.07 | 0.07 |
| U_C18:3 | -0.11 | -0.18 | -0.19 |
| U_C18:4 | 0.05 | -0.00 | 0.01 |

*, ** significant at the 0.05 and 0.01 level of the probability, respectively

Table S3. The properties of genetic linkage map of faba bean based on the BPP of Coˆte d’Or 1 (French landrace) and BPL 4628 (RILs mapping).

| Linkage group | Length (cM) | No. of markers | average interval |
| --- | --- | --- | --- |
| LG01 | 65.97 | 21 | 3.30 |
| LG02 | 53.81 | 18 | 3.17 |
| LG03 | 40.04 | 15 | 2.86 |
| LG04 | 29.61 | 21 | 1.48 |
| LG05 | 29.12 | 11 | 2.91 |
| LG06 | 24.76 | 7 | 4.13 |
| LG07 | 14.53 | 4 | 4.84 |
| LG08 | 12.46 | 5 | 3.11 |
| LG09 | 2.73 | 2 | 2.73 |
| LG10 | 4.11 | 3 | 2.06 |
| LG11 | 8.64 | 2 | 8.64 |
| LG12 | 5.94 | 3 | 2.97 |
| LG13 | 4.41 | 2 | 2.21 |
| LG14 | 1.03 | 3 | 0.52 |

Table S4: Localization of linkage groups of the current genetic map in the faba bean consensus map

| Faba bean consensus map (FBCM) ^(1)^ | | | Genetic linkage map (BPP) | | |
| --- | --- | --- | --- | --- | --- |
| FBCM | No. of markers | Length | LG | Length ^(2)^ | No. of markers ^(3)^ |
| FBCM_01 | 207 | 417.65 | LG02, LG07, LG09, LG11. LG14 | 80.73 | 29 (14.0%, 90.9%) |
| FBCM_02 | 135 | 230.62 | LG01 | 65.97 | 21 (15.5%, 90.5%) |
| FBCM_03 | 87 | 217.19 | LG05, LG12 | 35.07 | 14 (16.1%, 100%) |
| FBCM_04 | 100 | 209.87 | LG03, LG08 | 25.50 | 20 (20.0%, 100%) |
| FBCM_05 | 83 | 152.22 | LG06, LG10, LG13 | 32.99 | 12 (14.5%, 66.6%) |
| FBCM_06 | 75 | 176.2 | LG04 | 29.61 | 21 (28%, 80.9%) |

^(1)^ Webb et al. (2015).

^(2)^  Length of all linkage groups

^(3)^ No. of markers which do exist in the linkage groups of the genetic linkage map of BPP. The percentages in brackets refers to the percentage of markers (mapped in BPP) according to FBCM and of those which mapped in FBCM (e.g. 100% means that all markers in these linkage groups were mapped in FBCM), respectively.

Table S5: List of 54 SNP markers showing a promising genetic diversity with high polymorphic information content (PIC) and gene diversity (GD) values in the two different genetic backgrounds (GWBP and BPP).

| Marker name | PIC (GWBP) | PIC (BPP) | GD  (GWBP) | GD  (BPP) | LG^(1)^ | Pos. | *M. truncatula* Chro. ^(2)^ |
| --- | --- | --- | --- | --- | --- | --- | --- |
| Vf_Mt7g080730 | 0.37 | 0.37 | 0.50 | 0.50 | FBCM_01 | 0 | 7 |
| Vf_Mt2g098890 | 0.37 | 0.37 | 0.50 | 0.50 | FBCM_01 | 26.92 | 2 |
| Vf_Mt2g089140 | 0.36 | 0.37 | 0.47 | 0.49 | FBCM_01 | 58.13 | 2 |
| Vf_Mt2g086880 | 0.36 | 0.37 | 0.47 | 0.50 | FBCM_01 | 62.66 | 2 |
| Vf_Mt6g008170 | 0.37 | 0.37 | 0.49 | 0.50 | FBCM_01 | 131.02 | 6 |
| Vf_Mt5g078030 | 0.36 | 0.37 | 0.48 | 0.50 | FBCM_01 | 200.49 | 5 |
| Vf_Mt5g076620 | 0.37 | 0.37 | 0.50 | 0.50 | FBCM_01 | 202.92 | 5 |
| Vf_Mt5g033880 | 0.35 | 0.37 | 0.45 | 0.50 | FBCM_01 | 265.47 | 5 |
| Vf_Mt5g037120 | 0.37 | 0.37 | 0.50 | 0.50 | FBCM_01 | 271.72 | 5 |
| Vf_Mt5g044970 | 0.35 | 0.31 | 0.46 | 0.39 | FBCM_01 | 293.36 | 5 |
| Vf_Mt5g046030 | 0.37 | 0.37 | 0.50 | 0.50 | FBCM_01 | 293.36 | 5 |
| Vf_Mt5g013980 | 0.36 | 0.38 | 0.47 | 0.50 | FBCM_01 | 346.09 | 5 |
| Vf_Mt5g011550 | 0.36 | 0.37 | 0.47 | 0.49 | FBCM_01 | 352.47 | 5 |
| Vf_Mt5g009720 | 0.36 | 0.37 | 0.46 | 0.50 | FBCM_01 | 356.08 | 5 |
| Vf_Mt2g027240 | 0.37 | 0.37 | 0.50 | 0.49 | FBCM_01 | 401.92 | 2 |
| Vf_Mt2g015010 | 0.36 | 0.37 | 0.48 | 0.50 | FBCM_01 | 429.11 | 2 |
| Vf_Mt3g076590 | 0.35 | 0.37 | 0.45 | 0.50 | FBCM_02 | 83.97 | 3 |
| Vf_Mt3g077670 | 0.37 | 0.37 | 0.50 | 0.50 | FBCM_02 | 87.28 | 3 |
| Vf_Mt3g092310 | 0.36 | 0.37 | 0.47 | 0.50 | FBCM_02 | 121.83 | 3 |
| Vf_Mt3g100500 | 0.35 | 0.37 | 0.45 | 0.50 | FBCM_02 | 139.30 | 3 |
| Vf_Mt3g117120 | 0.36 | 0.37 | 0.47 | 0.50 | FBCM_02 | 193.05 | 3 |
| Vf_Mt4g014710 | 0.36 | 0.37 | 0.47 | 0.50 | FBCM_02 | 222.09 | 4 |
| Vf_Mt1g018320 | 0.37 | 0.38 | 0.49 | 0.50 | FBCM_03 | 13.9 | 1 |
| Vf_Mt1g017950 | 0.35 | 0.38 | 0.44 | 0.50 | FBCM_03 | 14.27 | 1 |
| REPSNP | 0.35 | 0.37 | 0.44 | 0.50 | FBCM_03 | 140.38 | - |
| Vf_Mt4g080370 | 0.37 | 0.38 | 0.49 | 0.50 | FBCM_03 | 232.16 | 4 |
| Vf_Mt3g117800 | 0.35 | 0.37 | 0.45 | 0.49 | FBCM_04 | 5.890 | 3 |
| Vf_Mt4g122670 | 0.37 | 0.37 | 0.50 | 0.50 | FBCM_04 | 48.65 | 4 |
| Vf_Mt4g114900 | 0.37 | 0.37 | 0.49 | 0.50 | FBCM_04 | 66.52 | 4 |
| Vf_Mt4g100760 | 0.37 | 0.37 | 0.49 | 0.50 | FBCM_04 | 111.36 | 4 |
| Vf_Mt8g076060 | 0.37 | 0.37 | 0.49 | 0.49 | FBCM_04 | 117.24 | 8 |
| Vf_Mt8g020800 | 0.36 | 0.37 | 0.48 | 0.50 | FBCM_04 | 141.28 | 8 |
| Vf_Mt8g040550 | 0.37 | 0.37 | 0.48 | 0.50 | FBCM_04 | 192.46 | 8 |
| Vf_Mt7g084010 | 0.36 | 0.37 | 0.47 | 0.50 | FBCM_05 | 147.47 | 7 |
| Vf_Mt7g110600 | 0.36 | 0.37 | 0.46 | 0.49 | FBCM_05 | 211.58 | 7 |
| Vf_Mt7g112640 | 0.36 | 0.37 | 0.48 | 0.50 | FBCM_05 | 224.09 | 7 |
| Vf_Mt7g113850 | 0.37 | 0.37 | 0.50 | 0.50 | FBCM_05 | 227.05 | 7 |
| Marker name | PIC (GWBP) | PIC (BPP) | GD  (GWBP) | GD  (BPP) | LG^(1)^ | Pos. | *M. truncatula* Chro. ^(2)^ |
| Vf_Mt7g116600 | 0.35 | 0.37 | 0.46 | 0.50 | FBCM_05 | 230.56 | 7 |
| New_GLIP307SNP | 0.36 | 0.37 | 0.47 | 0.49 | FBCM_06 | 15.95 | - |
| Vf_Mt8g102250 | 0.37 | 0.37 | 0.49 | 0.49 | FBCM_06 | 23.83 | 8 |
| Vf_Mt8g101390 | 0.37 | 0.37 | 0.50 | 0.49 | FBCM_06 | 29.60 | 8 |
| Vf_Mt8g092620 | 0.35 | 0.37 | 0.45 | 0.50 | FBCM_06 | 61.38 | 8 |
| Vf_Mt4g085890 | 0.37 | 0.37 | 0.50 | 0.50 | FBCM_06 | 86.86 | 4 |
| Vf_Mt4g085900 | 0.37 | 0.37 | 0.50 | 0.50 | FBCM_06 | 87.24 | 4 |
| Vf_Mt4g088010 | 0.38 | 0.37 | 0.50 | 0.50 | FBCM_06 | 95.89 | 4 |
| Vf_Mt4g068010 | 0.37 | 0.37 | 0.50 | 0.50 | FBCM_06 | 162.70 | 4 |
| Vf_Mt4g064820 | 0.37 | 0.37 | 0.49 | 0.50 | FBCM_06 | 163.66 | 4 |
| Vf_Mt3g087150 | 0.37 | 0.37 | 0.50 | 0.50 | LG02 | 18.53 | 3 |
| Vf_Mt4g010330 | 0.37 | 0.37 | 0.48 | 0.50 | LG02 | 63.32 | 4 |
| GLIP081SNP | 0.37 | 0.37 | 0.49 | 0.50 | LG03 | 21.99 | - |
| Vf_Mt4g092850 | 0.36 | 0.37 | 0.47 | 0.50 | LG03 | 26.53 | 4 |
| Vf_Mt6g071210 | 0.36 | 0.37 | 0.47 | 0.50 | LG08 | 7.71 | 6 |
| Vf_Mt7g030010 | 0.37 | 0.37 | 0.49 | 0.50 | LG12 | 0.00 | 7 |
| Vf_Mt7g118320 | 0.37 | 0.38 | 0.50 | 0.50 | LG14 | 24.76 | 7 |

^(1)^ FBCM: linkage group in the faba bean consensus map, LG: linkage group in the current faba bean genetic map (101 RILs).

^(2)^ Chromosomes of *Medicago truncatula*

Appendix 1. List of traits abbreviations mentioned in the current study.

| Abbreviation | Name | Unit | Population | Reference |
| --- | --- | --- | --- | --- |
| PBB | Bi-parental population | - | - | (Arbaoui et al., 2008) |
| GWBP | Gottingen winter bean population | - | - | (Sallam et al., 2015) |
| H_AUSPC | area under symptom progress curve after hardening | - | BPP | (Arbaoui et al., 2008) |
| U_AUSPC | area under symptom progress curve without hardening | - | BPP | (Arbaoui et al., 2008) |
| M_AUSPC | Mean of H_AUSPC and U_AUSPC | - | BPP | (Arbaoui et al., 2008) |
| H_C16:0 ^(1)^ | C16:0 after hardening | % | BPP | (Arbaoui et al., 2008) |
| H_C18:0 ^(1)^ | C18:0 after hardening | % | BPP | (Arbaoui et al., 2008) |
| H_C18:1 ^(1)^ | C18:1 after hardening | % | BPP | (Arbaoui et al., 2008) |
| H_C18:2 ^(1)^ | C18:2 after hardening | % | BPP | (Arbaoui et al., 2008) |
| H_C18:3 ^(1)^ | C18:3 after hardening | % | BPP | (Arbaoui et al., 2008) |
| H_C18:4 ^(1)^ | C18:4 after hardening | % | BPP | (Arbaoui et al., 2008) |
| U_C16:0 | C16:0 without hardening | % | BPP | (Arbaoui et al., 2008) |
| U_C16:1 | C16:1 without hardening | % | BPP | (Arbaoui et al., 2008) |
| H_AUSPC | area under symptom progress curve after hardening | - | GWBP | Current study |
| LTAF | Loss of leaf turgidity after frost | 1 - 9 | GWBP | Current study |
| LCAF | Loss of leaf color after frost | 1 - 9 | GWBP | Current study |
| DS | disposition to survive after frost | ° | GWBP | (Sallam et al., 2015) |
| I_1_ | index 1 (including DS) | Arbitrary units | GWBP | Sallam et al., 2015) |
| I_2_ | index 2 (including regrowth after frost) | Arbitrary units | GWBP | Sallam et al., 2015) |
| FTI | Frost tolerance index | Arbitrary units | GWBP | Sallam et al., 2015) |
| LT+LC | loss of color + loss of turgidity | 8 - 72 | GWBP | Sallam et al., 2015) |
| RDF | reduction in water content due to frost stress | % | GWBP | Sallam et al., 2015) |
| RWCAF | relative water content after frost | % | GWBP | Sallam et al., 2015) |
| FPH | field plant height | cm | GWBP | (Sallam et al., 2016) |
| SFA | saturated fatty acid content | % | GWBP | (Sallam et al., 2016) |
| DTF | days to flowering | days | GWBP | (Sallam et al., 2016) |

^(1)^ These traits were also analyzed in GWBP and previously published in (Sallam et al., 2015)
